# M^5^C-driven stabilization of SERPINB5 promotes cervical cancer progression and chemotherapy resistance

**DOI:** 10.1038/s41419-026-08453-2

**Published:** 2026-02-11

**Authors:** Jiejie Liu, Limin Zhou, Peipei Yao, Nan Zhang, Xiao Guo, Fei Chen, Shimin Yang, Xin Du, Hongyun Wang, You Zhou, Yu Chen, Li Zhou

**Affiliations:** 1 State Key Laboratory of Virology and Biosafety, RNA Institute, College of Life Sciences and Frontier Science Center for Immunology and Metabolism, Wuhan University, Wuhan, China; 2 Maternal and Child Health Hospital of Hubei Province, Tongji Medical College, Huazhong University of Science and Technology, Wuhan, China; 3 Animal Bio-safety Level III Laboratory/Institute for Vaccine Research, Taikang Medical School (School of Basic Medical Sciences), Wuhan University, Wuhan, China; 4 Biosafety Laboratory, lnternational Center for Biological and Translational Research, West China Hospital, Sichuan University, Chengdu, China; 5 Systems Immunity Research Institute, Cardiff University, Cardiff, UK; 6 Division of Infection and Immunity, School of Medicine, Cardiff University, Cardiff, UK

**Keywords:** Cancer, Molecular biology

## Abstract

RNA 5-methylcytosine (m^5^C) plays a critical role in cancer, yet its functional mechanisms and therapeutic relevance in cervical cancer remain unclear. Here, we generate the first base-resolution m^5^C transcriptome maps in cervical cancer, revealing globally elevated m^5^C levels in tumors. By integrating spatial transcriptomics and single-cell RNA-seq, we identify SERPINB5 as a novel m^5^C-regulated oncogenic effector. m^5^C modification enhances SERPINB5 mRNA stability and protein expression, promoting tumor growth, metastasis, and resistance to microtubule-targeting chemotherapeutics. Mechanistically, SERPINB5 upregulates mitotic regulators and microtubule motor proteins, including CENPE, enhancing mitotic progression and counteracting drug-induced mitotic arrest. Loss-of-function experiments demonstrate that SERPINB5 depletion sensitizes cervical cancer cells to paclitaxel and vincristine, while its reintroduction restores chemoresistance even in m^5^C-deficient cells. Our study uncovers a previously unrecognized m^5^C–SERPINB5 axis as a central driver of cervical cancer malignancy and chemoresistance, highlighting SERPINB5 as a clinically actionable target to improve outcomes for patients receiving microtubule-targeting chemotherapy.

## Introduction

Cervical cancer remains a major global health challenge, ranking as the fourth most common malignancy in women worldwide, with an estimated 660,000 new cases and over 350,000 deaths annually [1, 2]. Despite progress in prevention through human papillomavirus (HPV) vaccination and early detection via screening, the disease disproportionately affects women in low- and middle-income countries, where access to these advances remains limited [3, 4]. The clinical outcome for patients with advanced or recurrent cervical cancer remains poor, with 5-year survival rates below 17%, primarily due to resistance to frontline chemotherapy [5].

Paclitaxel-based regimens, often combined with platinum compounds, form the cornerstone of treatment for advanced cervical cancer [6]. However, chemoresistance, especially to microtubule-targeting agents such as paclitaxel and vincristine, remains a critical hurdle [7, 8]. Classical mechanisms of resistance, including upregulation of efflux pumps (e.g., MDR1), mutations in tubulin isoforms, and mitotic checkpoint alterations [9–12], explain resistance in some settings but fail to account for many clinical cases. These gaps have prompted efforts to uncover alternative, non-genetic resistance mechanisms, including those governed by post-transcriptional and epigenetic regulators [13]. Recent studies have highlighted RNA modifications as potent modulators of cancer progression and therapeutic response [14–18]. Among these, RNA 5-methylcytosine (m^5^C) represents a chemically stable and functionally diverse mark deposited by methyltransferases such as NSUN2 and interpreted by m^5^C-binding proteins like YBX1 [19–21]. m^5^C influences RNA metabolism at multiple levels, including stability, translation, and localization [19, 22]. NSUN2 is frequently overexpressed in aggressive tumors and has been implicated in stress adaptation, metastasis, and therapy resistance in various cancers [23–25]. However, the role of NSUN2-driven m^5^C signaling in cervical cancer, particularly its contribution to resistance against microtubule-targeting chemotherapy, remains unexplored [26, 27]. Moreover, the downstream effectors mediating these phenotypes have yet to be defined.

SERPINB5, also known as maspin, is a non-inhibitory member of the serine protease inhibitor superfamily originally identified as a tumor suppressor in breast cancer [28]. Subsequent studies have revealed that SERPINB5 exhibits highly context-dependent roles across cancer types. While loss of SERPINB5 expression correlates with tumor progression and metastasis in breast and prostate cancers [29–32], its elevated expression in colorectal and lung cancers is associated with tumor progression and poor prognosis [33, 34]. In nasopharyngeal carcinoma, SERPINB5 interacts with TRIM21 to inhibit GMPS, thereby protecting cells from radiation-induced apoptosis [35]. It also serves as a biomarker of radioresistance in triple-negative breast cancer [36]. A recent study identified a SERPINB5/TMEM45A/p16INK4a signature as a marker for high-grade cervical lesions [37]. Notably, germline variants in SERPINB5 have been linked to an increased predisposition to various cancers [38–41]. Despite these findings, the mechanisms regulating SERPINB5 expression remain unclear, particularly at the post-transcriptional level, including regulation via RNA methylation.

In this study, we present the first base-resolution m^5^C methylome of cervical cancer, integrating RNA bisulfite sequencing (BS-seq) with spatial transcriptomics (ST) and single-cell RNA sequencing (scRNA-seq). We identify SERPINB5 as a novel m^5^C-regulated oncogenic effector. NSUN2-dependent m^5^C modification enhances *SERPINB5* mRNA stability and protein expression, promoting tumor growth, metastasis, and resistance to paclitaxel and vincristine. Mechanistically, SERPINB5 upregulates mitotic regulators such as CENPE and sustains mitotic progression, counteracting the cytotoxic effects of microtubule inhibitors. Importantly, both in vitro and in vivo loss-of-function assays demonstrate that targeting the NSUN2*–*SERPINB5 axis sensitizes tumors to chemotherapy, while re-expression of SERPINB5 restores resistance in m^5^C-deficient cells. Collectively, our findings reveal an epitranscriptomic mechanism driving chemoresistance through m^5^C-mediated stabilization of *SERPINB5* and demonstrate this pathway as a promising therapeutic target in cervical cancer.

## Results

### Global m^5^C hypermethylation reprograms oncogenic pathways in cervical cancer

**Fig. 1:**
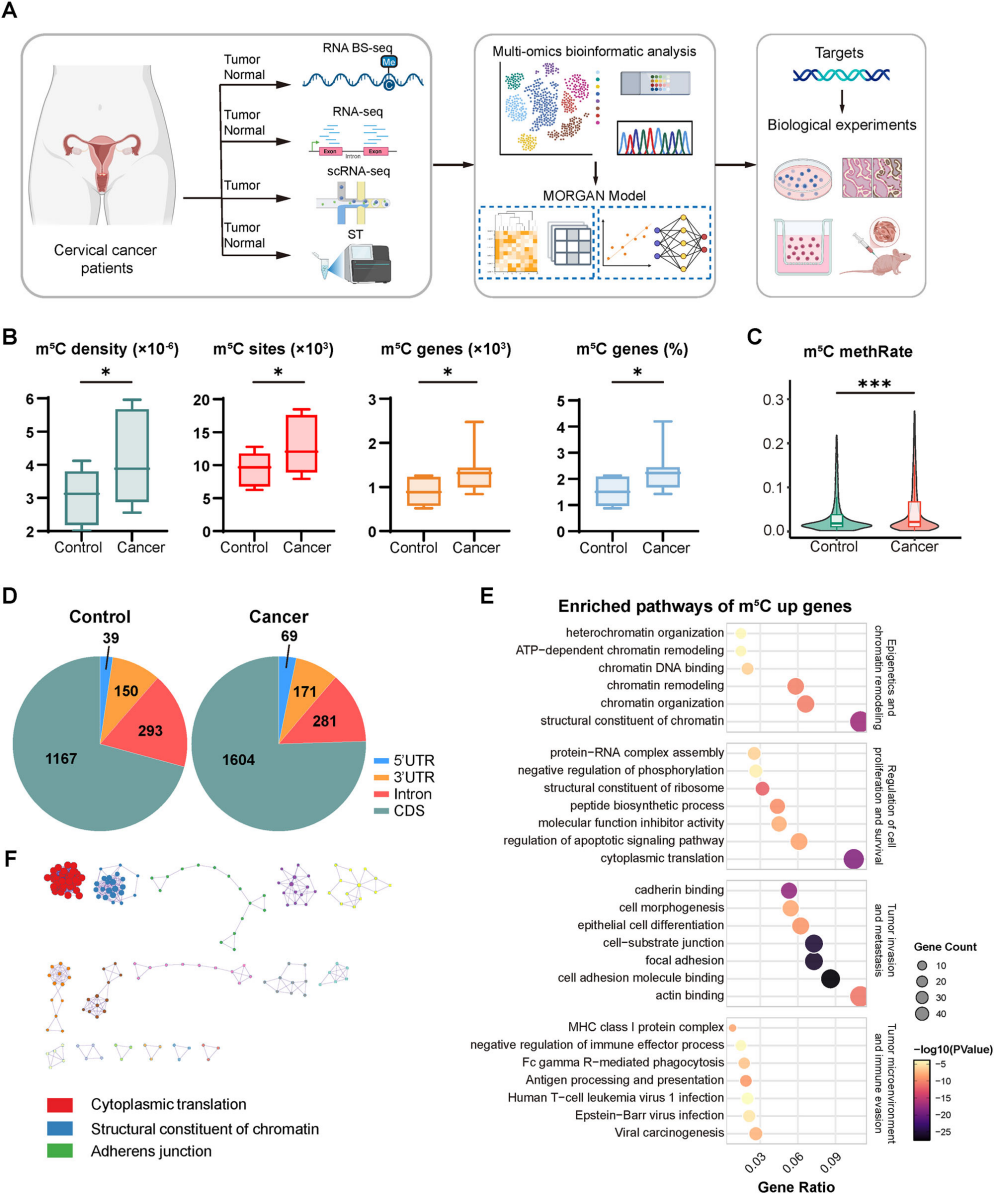
Global RNA m^5^C hypermethylation and its functional implications in cervical cancer. **A** Schematic overview of the study design. Tumor and normal cervical tissues were analyzed using RNA bisulfite sequencing (RNA BS-seq), bulk RNA-seq, and spatial transcriptomics (ST), while single-cell RNA-seq (scRNA-seq) was performed exclusively on tumor samples. The multi-omics data were integrated to construct the MORGAN model for target identification, followed by experimental validation. Created with BioRender. **B** Comparison of m^5^C density, number of m^5^C sites, number of m^5^C-modified genes, and percentage of m^5^C-modified genes between control (*n* = 8) and cancer (*n* = 14) samples. Data are presented as box plots showing the median and min to max range. Data were analyzed using a two-tailed unpaired t test. *P < 0.05. **C** Violin plots show the distribution and median of methylation rates across samples. The global per-site m^5^C methylation rate (methRate) was significantly elevated in cancer samples compared to controls (***P < 0.001, Wilcoxon rank-sum test). **D** Genomic distribution of m^5^C sites in control and cancer samples. Pie charts show the distribution of m^5^C sites across different transcript regions, including 5’UTR, 3’UTR, introns, and CDS. **E** Functional enrichment analysis of genes with significantly increased m^5^C methylation in cervical cancer. The Dot plot shows selected enriched terms and pathways grouped into four categories: Epigenetics and chromatin remodeling, Regulation of cell proliferation and survival, Tumor invasion and metastasis, and Tumor microenvironment and immune evasion. Dot size indicates the number of genes involved, and color represents the significance level. **F** A PPI network was constructed based on genes with significantly elevated m^5^C levels in cancer samples. Three major functional modules were identified using the MCODE algorithm.

To systematically characterize the epitranscriptomic landscape of cervical cancer, we performed RNA BS-seq on 14 primary cervical tumors and 8 normal cervical tissues. To capture potential intratumoral heterogeneity and enhance the robustness of m^5^C methylation profiling, multiple samples from spatially distinct regions were collected for some individual tumors (Fig. 1A and Table S1). The m^5^C profiles were highly consistent within each group, supporting the reliability of methylation quantification (Fig. S1A, B). Strikingly, we observed a widespread m^5^C hypermethylation in all cervical tumor samples. Up to 18,475 m^5^C sites were detected in a single tumor sample, with the number of sites across cancer samples ranging from 7925 to 18,475, substantially exceeding that observed in normal controls (6237 to 12,770) (Table S2). Consistently, tumor samples harbored more methylated genes (up to 2,474), along with increased density and methylation rate per transcript (Fig. 1B, C and Table S2). Distribution analysis revealed that m^5^C modifications were predominantly enriched within coding sequences (CDS), suggesting selective functional remodeling (Fig. 1D).

Overrepresentation analysis of hypermethylated genes revealed significant enrichment in biological processes related to epigenetic regulation and chromatin remodeling, as well as pathways governing cell proliferation, survival, invasion, metastasis, and immune escape (Fig. 1E and Table S3). To further explore functional relationships among these genes, we constructed a protein-protein interaction (PPI) network analysis and applied the MCODE algorithm. This revealed three major functional modules enriched in pathways related to cytoplasmic translation, structural constituents of chromatin, and adherens junctions. Adherens junctions and associated adhesion complexes transmit mechanical and biochemical signals from the membrane to the nucleus, potentially influencing chromatin architecture and gene expression [42]. Together with translational control in the cytoplasm, these modules form an interconnected network of fundamental processes implicated in tumor growth (Fig. 1F). These findings indicate that m^5^C hypermethylation is a pervasive and coordinated epitranscriptomic reprogramming event that targets core oncogenic pathways in cervical cancer.

### Integrated RNA-seq and m^5^C profiling identifies SERPINB5 as a candidate m^5^C-regulated oncogene

To evaluate whether m^5^C modifications coincide with transcriptional dysregulation in cervical cancer, we performed RNA-seq on 9 tumor and 7 normal cervical samples (Fig. 1A and Table S1). Despite incomplete overlap with the RNA BS-seq dataset, the available matched sets enabled robust integrative analysis. RNA expression profiles were highly consistent within each group, supporting the reliability of quantification and downstream comparisons (Fig. S1C, D).

**Fig. 2:**
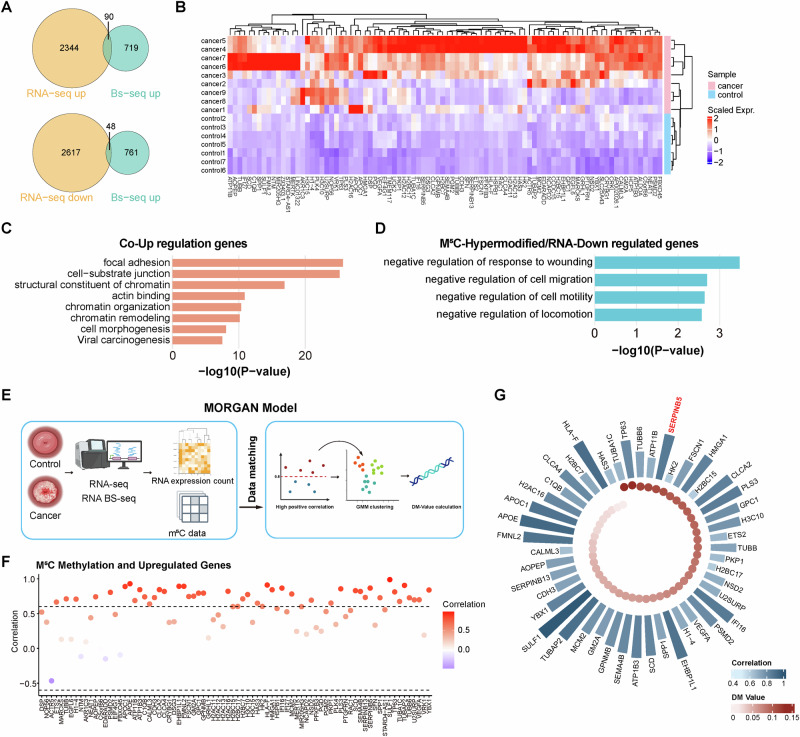
Transcriptome-wide integration of m^5^C methylation and gene expression identifies regulatory m^5^C targets in cervical cancer. **A** Venn diagrams showing the overlap between genes significantly upregulated (top) or downregulated (bottom) in RNA-seq and genes with increased m^5^C methylation detected by BS-seq in cancer compared to control samples. **B** Heatmap of scaled RNA expression levels of genes with concurrent upregulation and m^5^C hypermethylation across cancer and control samples. Expression values are scaled by Z-score. **C** Enrichment analysis of the co-upregulated genes. **D** Enrichment analysis of m^5^C-hypermethylated but RNA-downregulated genes. **E** Schematic of the MORGAN workflow. **F** Correlation plot between m^5^C methylation and RNA expression across genes that are upregulated in cancer. Each dot represents a gene, and dot color represents the correlation strength. **G** Blue bars represent the correlation between m^5^C methylation and gene expression, with greater height and deeper blue indicating stronger correlation. Red dots indicate DM values, with deeper red denoting higher values. Gene names are labeled around the ring.

Integrated analysis of BS-seq and RNA-seq data identified 90 genes with both elevated m^5^C methylation and upregulated expression in tumors, including several oncogenes such as *NEAT1*, *GPC1*, and *VEGFA* [43–45] (Fig. 2A, B). These genes were enriched in focal adhesion and cell-substrate junction organization, features often associated with enhanced cell motility and invasive potential [46]. Focal adhesions also serve as signaling hubs, conveying mechanical and biochemical cues to the nucleus, where they may influence chromatin remodeling, organization, and structural components. These genes are additionally involved in actin binding, cell morphogenesis, and viral carcinogenesis, highlighting a coordinated network linking cytoskeletal dynamics, epigenetic regulation, and HPV-driven oncogenic processes (Fig. 2C). Together, these findings indicate that m^5^C hypermethylation may promote tumor progression through coordinated regulation of cell adhesion, chromatin state, and structural remodeling. Conversely, 48 genes displayed elevated m^5^C levels but reduced expression in tumors, implying that m^5^C may also exert gene-silencing effects in specific contexts (Fig. 2A). These genes were enriched in biological processes associated with suppression of cellular movement, including negative regulation of cell migration, motility, locomotion, and response to wounding (Fig. 2D).

To systematically prioritize functional m^5^C effectors, we developed MORGAN (*M*^*5*^*C-Oriented RNA Gene Identification via Adaptive Gaussian Mixture Network*), a new machine learning-based framework integrating methylation and transcriptome data to identify regulatory targets with high tumor-specific m^5^C activation scores (Fig. 2E; see Methods). While a subset of genes showed increased methylation accompanied by reduced expression, none met the threshold for strong negative correlation across the transcriptome (Fig. S2), suggesting that m^5^C-mediated gene silencing may occur through indirect or context-dependent mechanisms. Therefore, we focused on positively correlated transcripts as candidate m^5^C-activated regulators (Fig. 2F). Based on the Differential Methylation (DM) value ranking, MORGAN identified top ten candidates: *TUBA1C*, *TP63*, *TUBB6*, *ATP11B*, *SERPINB5*, *HK2*, *FSCN1*, *HMGA1*, *H2BC15*, and *CLCA2* (Fig. 2G). Notably, several of these genes are well-established oncogenes. For instance, TP63 is a master regulator of epithelial lineage specification, while HK2 encodes a key metabolic enzyme central to glycolytic reprogramming in cancer cells [47–49]. These results demonstrate the ability of MORGAN to identify biologically meaningful targets by leveraging transcriptome-wide m^5^C methylation patterns. Among these candidates, *SERPINB5* exhibited the highest positive correlation between m^5^C methylation and gene expression (*r* = 0.87), highlighting a potential link between m^5^C modification and transcriptional activation (Fig. 2G).

### NSUN2 upregulates SERPINB5 through an m^5^C-dependent mechanism

**Fig. 3:**
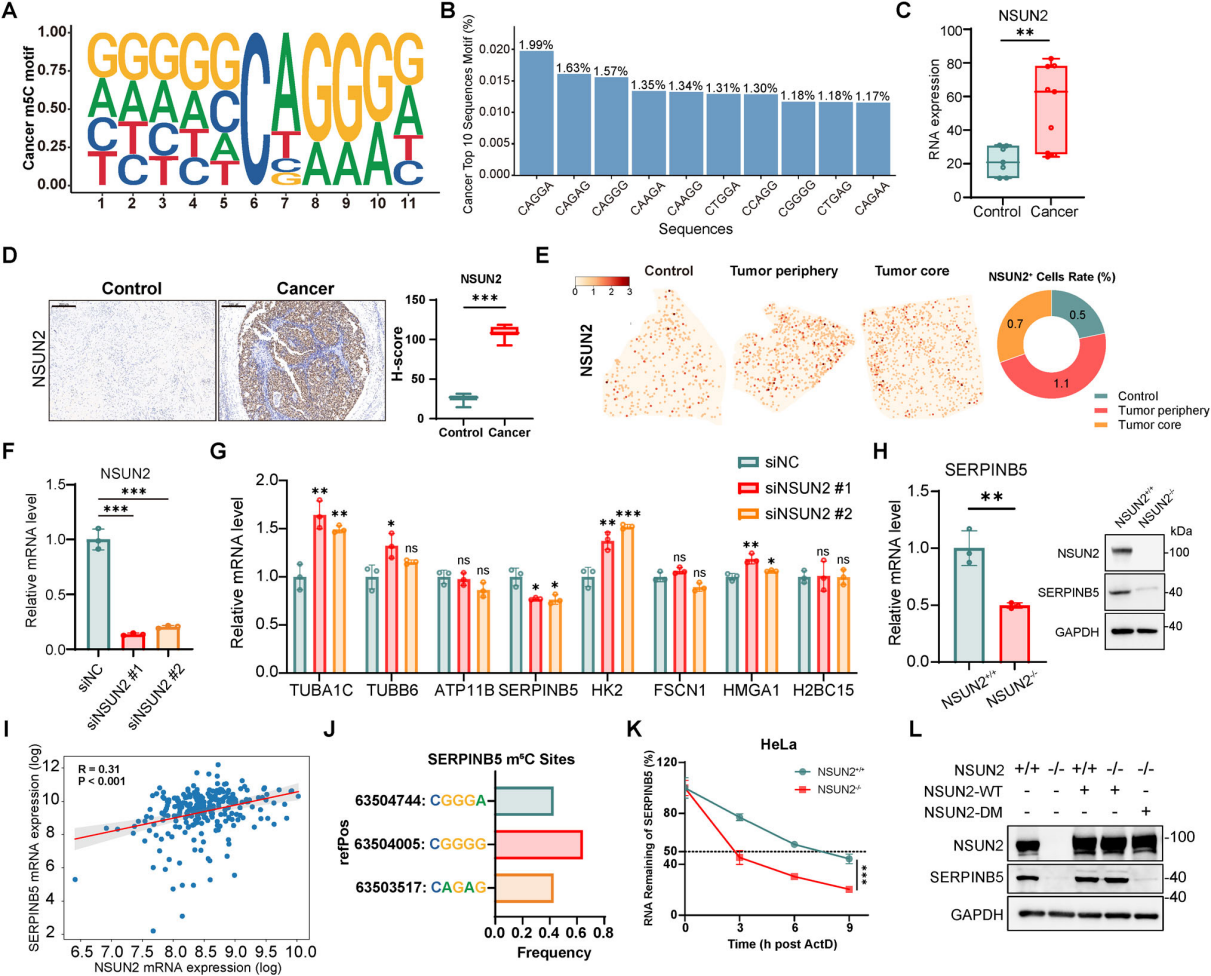
NSUN2 is upregulated in cervical cancer and promotes SERPINB5 expression. **A** Sequence motif enriched at m^5^C sites identified in cervical cancer samples. **B** Top 10 enriched sequence contexts flanking cancer-associated m^5^C sites. The percentage indicates the fraction of total m^5^C sites matching each motif. **C** Box plots show *NSUN2* expression levels measured by RNA-seq in control (*n* = 7) and cervical cancer (*n* = 9) samples. Data are presented as box plots showing the median and min to max range. **D** Representative immunohistochemical staining of NSUN2 in control and cervical cancer tissues (left). Scale bars, 200 μm. Quantification by H-score shows significantly higher NSUN2 protein level in cancer tissues (*n* = 8 per group) (right). Data are presented as box plots showing the median and min to max range. **E** Representative spatial mapping of *NSUN2* expression in control, tumor periphery, and tumor core. The pie chart shows the percentage of *NSUN2*^+^ cells. **F** qPCR analysis of *NSUN2* mRNA levels after siRNA-mediated knockdown (siNSUN2 #1 and siNSUN2 #2) in HeLa cells (*n* = 3). Data are presented as mean ± SD. **G** Relative mRNA expression of selected candidate genes following *NSUN2* knockdown (*n* = 3). Data are presented as mean ± SD. **H** *SERPINB5* mRNA level (left, *n* = 3) and protein levels (right) in wild-type (WT, NSUN2^+/+^) or NSUN2^–/–^ HeLa cells. For qPCR, data are presented as mean ± SD. **I** Correlation between *NSUN2* and *SERPINB5* mRNA expression in cervical cancer. **J** m^5^C-modified sites in *SERPINB5* transcripts identified by BS-seq. Sequences and modification frequencies of the m^5^C sites are shown. **K** RNA stability assay of *SERPINB5* mRNA after actinomycin D treatment in NSUN2^+/+^ or NSUN2^–/–^ HeLa cells. Remaining mRNA was quantified by qPCR at the indicated time points (*n* = 3). Data are presented as mean ± SD. **L** Immunoblot analysis of protein levels of SERPINB5 in NSUN2^+/+^ or NSUN2^–/–^ Hela cells. Cells were transfected with WT NSUN2 (NSUN2-WT) or I302A/C321A double mutation (NSUN2-DM). Statistical analyses were performed using the two-tailed unpaired t test for (**C**, **D**-right, **F**, **G**, **H**-left), Pearson correlation for association analysis for (**I**), and two-way ANOVA followed by multiple comparisons test for (**K**). NS not significant for *P* > 0.05, *P < 0.05, **P < 0.01, ***P < 0.001.

To elucidate the mechanism by which m^5^C promotes SERPINB5 upregulation, we first sought to identify the major m^5^C writer responsible for this modification. Motif analysis of the m^5^C sites identified by BS-seq revealed a highly conserved pattern in both tumor and normal tissues, characterized by a methylated cytosine followed by a G-rich triplet motif (3’NGGG) (Figs. 3A and S3A). Representative examples include CAGGG, CGGGG, CCAGG, and related variants (Figs. 3B and S3B). This flexible yet consistent G-rich motif is a known signature of NSUN2-dependent m^5^C methylation targets [50], strongly implicating NSUN2 as the predominant methyltransferase responsible for m^5^C deposition in cervical epithelial tissues. These findings align with previously reported NSUN2 motif preferences in HeLa cells and other human cell lines [50, 51].

To define NSUN2 expression patterns in cervical cancer, we integrated bulk RNA-seq, ST from our previous work [52], and a public scRNA-seq dataset [53]. NSUN2 was markedly upregulated in tumor tissues compared to normal samples across multiple datasets (Figs. 3C and S3C; Table S4) and was confirmed at the protein level by IHC staining (Fig. 3D). ST data showed that *NSUN2*^+^ cells were spatially enriched at the tumor periphery, followed by the core and normal regions (Figs. 3E and S3D), suggesting a potential role in tumor-stroma interactions. scRNA-seq further revealed *NSUN2* expression across diverse cell populations, including cancer cells (Fig. S3E, F). *NSUN2*⁺ cells displayed transcriptional programs characterized by enrichment in cell cycle-related pathways (e.g., positive regulation of cell cycle, DNA repair) and metabolic reprogramming (Fig. S3G and Table S5).

Given these observations, we hypothesized that NSUN2 may exert its oncogenic function by modulating m^5^C on specific downstream targets involved in tumor progression. We further examined the expression of the top MORGAN-prioritized genes following NSUN2 knockdown (Fig. 3F). Among these, only *SERPINB5* expression significantly declined, suggesting a unique dependence on NSUN2 (Fig. 3G). This downregulation was further validated in NSUN2 knockout (NSUN2^–/–^) HeLa cells and SiHa cells, indicating a dose-dependent regulatory effect (Figs. 3H and S3H). A positive correlation between *NSUN2* and *SERPINB5* expression in TCGA cervical cancer samples further supported their functional relationship (Fig. 3I).

To determine if *SERPINB5* is a direct substrate of NSUN2-catalyzed m^5^C methylation, we examined three m^5^C sites within its transcript (located at genomic coordinates 63504744 [CGGGA], 63504005 [CGGGG], and 63503517 [CAGAG]), all matching the G-rich motif associated with NSUN2 activity (Fig. 3J). NSUN2 knockout led to a marked reduction in *SERPINB5* mRNA stability (Figs. 3K and S3I). We next tested whether this effect required NSUN2 catalytic activity by overexpressing either wild-type NSUN2 or catalytically inactive NSUN2 double mutant (I302A/C321A) in NSUN2^–/–^ HeLa cells. Only wild-type NSUN2 restored *SERPINB5* mRNA and protein levels, whereas the mutant failed to rescue expression, confirming that the m^5^C methyltransferase activity of NSUN2 is required for maintaining SERPINB5 expression (Fig. 3L). Collectively, these findings demonstrate that NSUN2 catalyzes m^5^C methylation at specific cytosine residues within the *SERPINB5* transcript, thereby enhancing its stability and promoting its expression.

### YBX1 recognizes NSUN2-deposited m^5^C to mediate SERPINB5 mRNA stabilization

**Fig. 4:**
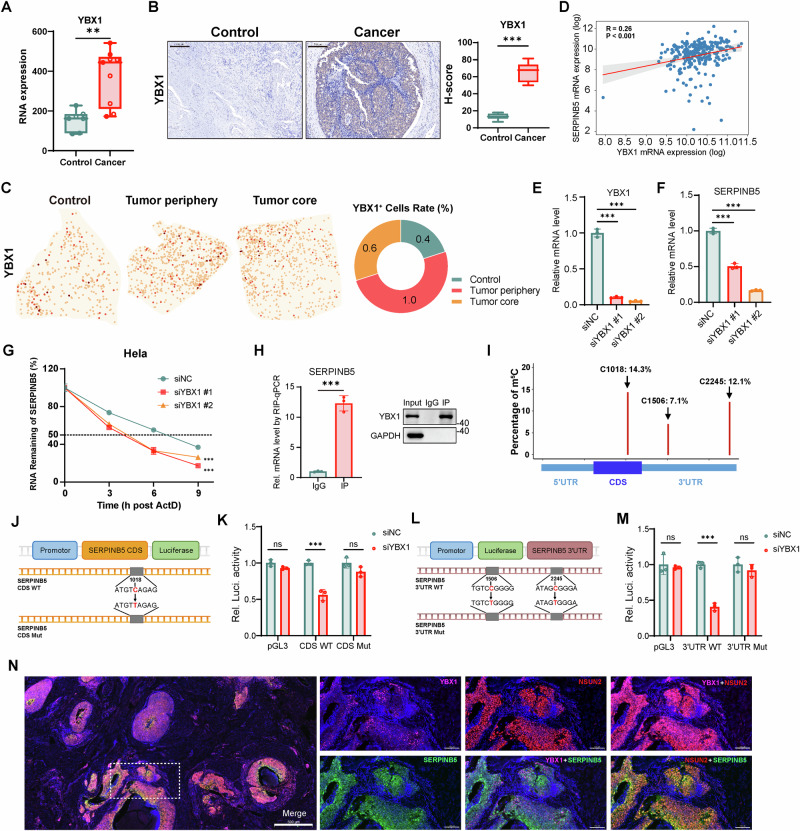
YBX1 recognizes NSUN2-deposited m^5^C to promote SERPINB5 mRNA stability and expression in cervical cancer. **A** Box plots showing *YBX1* expression levels measured by RNA-seq in control (*n* = 7) and cervical cancer (*n* = 9) samples. **B** Representative immunohistochemical staining of YBX1 in control and cervical cancer tissues (left). Scale bars, 200 μm. Quantification by H-score shows significantly higher YBX1 protein levels in cancer tissues (*n* = 8 per group) (right). Data are presented as box plots showing the median and min to max range. **C** Representative spatial mapping of *YBX1* expression in control, tumor periphery, and tumor core. The pie chart quantifies the percentage of *YBX1*^+^ cells. **D** Correlation between *YBX1* and *SERPINB5* mRNA expression in cervical cancer samples. **E** qPCR analysis of *YBX1* mRNA levels after siRNA-mediated knockdown (siYBX1 #1 and siYBX1 #2) in HeLa cells (*n* = 3). Data are presented as mean ± SD. **F** Relative expression of *SERPINB5* mRNAs following *YBX1* knockdown in HeLa cells (*n* = 3). Data are presented as mean ± SD. **G** RNA stability assay of *SERPINB5* mRNA in HeLa cells treated with actinomycin D following negative control (siNC) or *YBX1* siRNA knockdown. Remaining mRNA levels were quantified by qPCR at the indicated time points (*n* = 3). Data are presented as mean ± SD. **H** YBX1-RIP-qPCR analysis was performed using an anti-YBX1 antibody to immunoprecipitate HA-tagged YBX1, and the enrichment of *SERPINB5* mRNA was subsequently assessed (left) (*n* = 3). Data are presented as mean ± SD. Immunoblot analysis confirmed the presence of YBX1 protein in both input and immunoprecipitated (IP) samples, with IgG used as a negative control (right). **I** Schematic illustration of the three predominant m^5^C methylation sites identified within *SERPINB5* transcripts, including one site located in the CDS and two sites within the 3’UTR, along with their respective methylation frequencies. **J** Schematic of the SERPINB5 CDS luciferase reporter containing a single m^5^C site mutation (C1018T). **K** HeLa cells were transfected with pGL.3.0-CMV-Luc or pGL3.0-CMV-SERPINB5-CDS-Luc (WT or C1018T mutant), together with Renilla luciferase (RL-TK), followed by treatment with *YBX1* siRNA or control siRNA treatment. Luciferase activity was measured 48 h later, and firefly luciferase activity was normalized to Renilla luciferase activity (*n* = 3). Data are presented as mean ± SD. **L** Schematic of the SERPINB5 3’UTR luciferase reporter containing double m^5^C site mutations (C1506T/C2245T). **M** HeLa cells were transfected with pGL.3.0-CMV-Luc or pGL3.0-CMV-SERPINB5-3’UTR-Luc (WT or double mutant), together with RL-TK, and treated with *YBX1* siRNA or control siRNA. Firefly luciferase activity was measured and normalized to Renilla luciferase activity 48 h post-transfection (*n* = 3). Data are presented as mean ± SD. **N** Immunofluorescence microscopy of cervical cancer tissues stained with DAPI (blue), YBX1 (purple), SERPINB5 (green), and NSUN2 (red) antibodies. Left: merged image showing co-localization (scale bar, 500 μm). Right: individual and pairwise merged images showing distribution patterns of NSUN2, YBX1, and SERPINB5 (scale bar, 100 μm). Statistical analyses were performed using two-tailed unpaired t test for (**A**, **B**-right, **E**, **F**, **H**-left, **K**, **M**), Pearson correlation for association analyses (**D**), and two-way ANOVA followed by multiple comparisons test for (**G**). NS not significant for *P* > 0.05, *P < 0.05, **P < 0.01, ***P < 0.001.

To systematically identify potential m^5^C reader mediating the functional consequences of NSUN2-dependent *SERPINB5* methylation, we firstly examined the expression of five previously reported m^5^C-binding proteins in cervical cancer samples. Among these, YBX1 exhibited the most pronounced upregulation, despite not being the most abundantly expressed (Figs. 4A and S4A; Table S4). Immunohistochemical staining confirmed a significant increase of YBX1 protein in cervical cancer tissues compared to normal controls (Fig. 4B). By integrating ST and scRNA-seq data, we observed broad distribution of *YBX1* across the tumor architecture and various cell types (Figs. 4C and S4B). Notably, *YBX1*^+^ cells, similar to *NSUN2*^+^ cells, were predominantly localized at the tumor periphery, with a gradual decrease in abundance toward the tumor core and normal regions (Fig. 4C).

To investigate the functional role of *YBX1*^+^ cells, we performed differential expression analysis between *YBX1*^+^ and *YBX1*^-^ cells, followed by functional enrichment analysis (Table S6). *YBX1*^+^ cells exhibited significant upregulation of genes involved in cell cycle progression (e.g., regulation of metaphase/anaphase transition of cell cycle, cell cycle checkpoint signaling, mitotic spindle assembly checkpoint signaling, and DNA replication), and mitochondrial metabolism (e.g., oxidative phosphorylation, TCA cycle, and 2-oxocarboxylic acid metabolism) (Fig. S4C). Consistently, analysis of TCGA cervical cancer datasets revealed a significant positive correlation between *YBX1* and *SERPINB5* expression levels (Fig. 4D). Given that YBX1 stabilizes m^5^C-modified transcripts [20], we hypothesized that YBX1 acts as an m^5^C reader for *SERPINB5*.

To determine whether YBX1 regulates SERPINB5 expression, we silenced YBX1 in HeLa and SiHa cells using siRNAs and assessed endogenous *SERPINB5* transcript levels (Figs. 4E and S4D). YBX1 knockdown resulted in a significant decrease in *SERPINB5* mRNA abundance in both cell lines (Figs. 4F and S4E). In addition, mRNA stability assays following actinomycin D treatment showed that YBX1 knockdown markedly accelerated *SERPINB5* transcript decay, suggesting that YBX1 contributes to the post-transcriptional stabilization of *SERPINB5* (Figs. 4G and S4F). RIP-qPCR assays using a YBX1-specific antibody confirmed a significant enrichment of *SERPINB5* mRNA in YBX1-immunoprecipitated fractions (Fig. 4H).

To identify whether YBX1-mediated regulation of *SERPINB5* depends on m^5^C methylation, we identified three major m^5^C sites within the *SERPINB5* transcript, including one in the CDS and two in the 3’ untranslated region (3’UTR) (Fig. 4I). To assess the functional relevance of the CDS methylation site, we generated luciferase reporter constructs containing either the wild-type SERPINB5 CDS or a methylation-deficient mutant in which the C1018 site was substituted with T (C1018T) (Fig. 4J). In cells expressing the wild-type construct, YBX1 depletion led to a significant reduction in luciferase activity, whereas the activity of the mutant construct remained largely unchanged (Fig. 4K). We next examined whether YBX1 also regulates *SERPINB5* via m^5^C sites in the 3’UTR. To this end, we generated luciferase reporters containing either the wild-type 3’UTR or a double mutant in which both C1506 and C2245 were replaced with T (C1506T/C2245T) (Fig. 4L). YBX1 knockdown significantly reduced luciferase activity in the wild-type reporter, while the double mutant remained unaffected (Fig. 4M). To validate these findings at the spatial level, we performed immunofluorescence staining and observed strong spatial co-localization of NSUN2, YBX1, and SERPINB5 proteins in cervical cancer tissue (Fig. 4N). Taken together, these data support a model in which YBX1 recognizes NSUN2-deposited m^5^C marks on *SERPINB5* transcripts to promote their stability and expression.

### SERPINB5^+^ cancer cells define a transcriptionally distinct and spatially enriched malignant subpopulation in cervical cancer

**Fig. 5:**
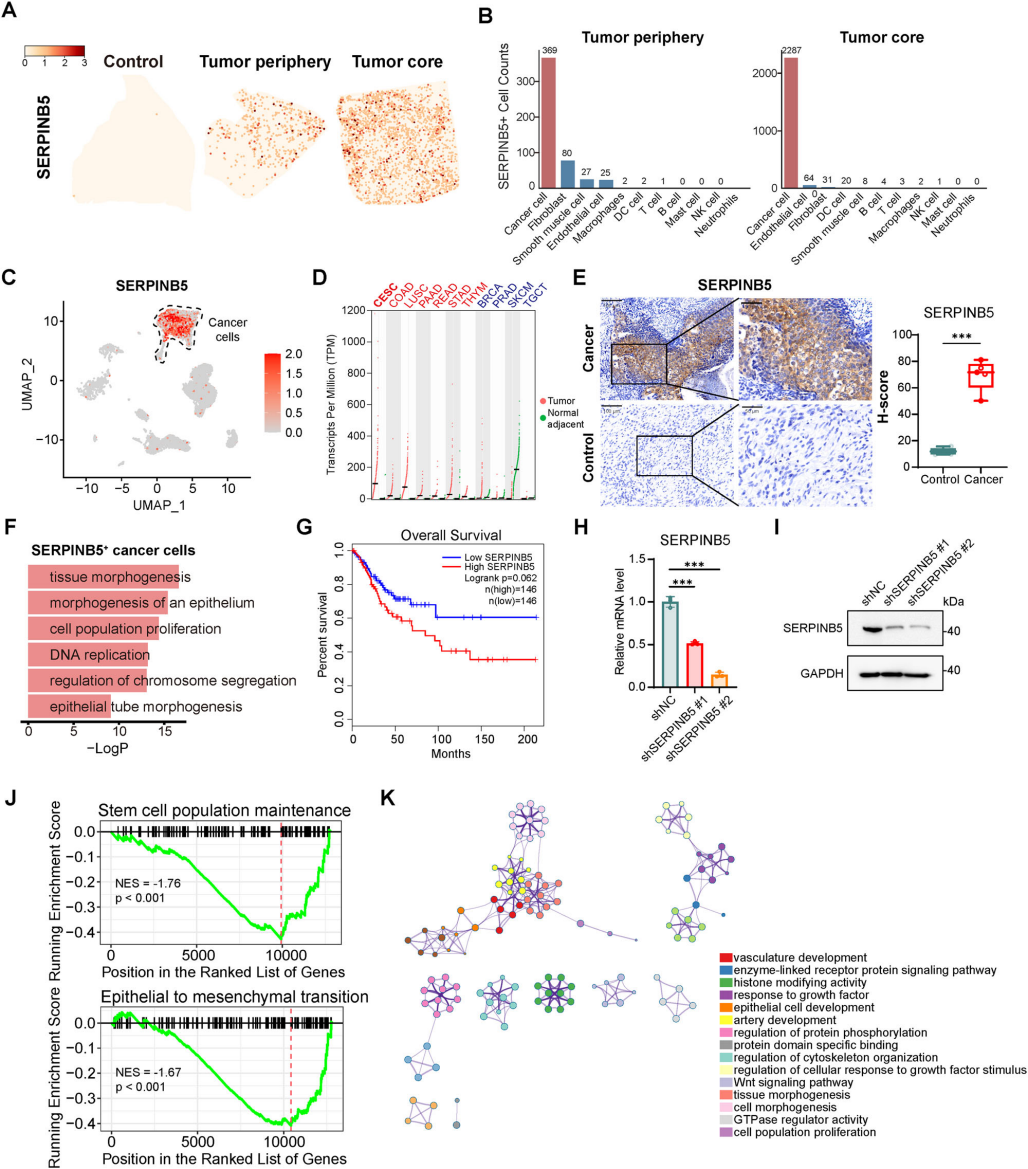
Multi-level analyses identify SERPINB5^+^ cancer cells as a malignant subpopulation in cervical cancer. **A** Representative spatial mapping of *SERPINB5* expression in control, tumor periphery, and tumor core. **B** Quantification of *SERPINB5*⁺ cell numbers across major cell types in tumor periphery and core. Cancer cells accounted for the majority of *SERPINB5*⁺ cells in both regions. **C** UMAP plot showing *SERPINB5* expression across single cells from tumor samples. Expression was predominantly restricted to cancer cells. **D** *SERPINB5* transcript levels across tumor and matched normal tissues in TCGA pan-cancer cohorts. Red dots indicate tumor samples; green dots indicate normal samples. **E** Representative immunohistochemical staining of SERPINB5 in control and cervical cancer tissues (left) Scale bars, 100 μm (low magnification) and 50 μm (high magnification). Quantification by H-score shows significantly higher SERPINB5 protein levels in cancer tissues (*n* = 5 per group) (right). Data are presented as box plots showing the median and min to max range. **F** Enrichment analysis of genes upregulated in *SERPINB5*⁺ cancer cells (vs. *SERPINB5*⁻). **G** Kaplan–Meier analysis of overall survival in TCGA cervical cancer cohort stratified by *SERPINB5* expression levels. **H** qPCR analysis showing *SERPINB5* mRNA levels in HeLa cells transfected with shNC, shSERPINB5 #1, or shSERPINB5 #2 (*n* = 3). Data are presented as mean ± SD. **I** Immunoblot analysis of SERPINB5 protein levels in HeLa cells shown in (**H**). **J** Gene set enrichment analysis (GSEA) showing suppression of stem cell maintenance and epithelial to mesenchymal transition programs upon SERPINB5 knockdown. Normalized enrichment score (NES) and P-values are shown. **K** Network representation of significantly downregulated signaling pathways enriched upon SERPINB5 knockdown. Statistical significance for comparisons in **E**-right and **H** was determined using the two-tailed unpaired t test. NS not significant for *P* > 0.05, *P < 0.05, **P < 0.01, ***P < 0.001.

Having established *SERPINB5* as a downstream effector stabilized by the NSUN2/YBX1-mediated m^5^C modification axis, we next sought to elucidate its functional role in cervical cancer. ST data analysis revealed negligible *SERPINB5* expression in normal cervical tissues, while tumor samples displayed markedly elevated expression localized to cancer cell-enriched regions (Fig. 5A). Notably, *SERPINB5*^+^ cells were most abundant among cancer cells within the tumor (Fig. 5B). This spatial enrichment was corroborated by scRNA-seq, which confirmed that *SERPINB5* expression was restricted to a specific subset of cancer cells, with minimal expression in immune or stromal compartments (Figs. 5C and S3E). Pan-cancer analysis of TCGA data revealed marked heterogeneity in *SERPINB5* expression across tumor types (Fig. 5D). Among these, cervical cancer exhibited one of the highest *SERPINB5* expression levels, along with other squamous and gastrointestinal malignancies such as lung, colon, and stomach cancers. In contrast, expression was lower in hormone-driven and melanocytic tumors. These findings suggest a context-dependent role of SERPINB5, with potential oncogenic activity in specific tumor lineages such as cervical cancer. Supporting the transcriptomic data, immunohistochemical analysis confirmed elevated SERPINB5 protein levels in cervical cancer tissues compared to matched normal counterparts (Fig. 5E).

To characterize the gene expression landscape of *SERPINB5*-expressing cells, we performed differential gene expression analysis between *SERPINB5*^+^ and *SERPINB5*^-^ cancer cells (Table S7). *SERPINB5*^+^ cells are transcriptionally distinct, characterized by strong enrichment of proliferative and morphogenetic programs, including DNA replication, chromosome segregation, epithelial morphogenesis, and epithelial tube formation (Fig. 5F). These results indicate that *SERPINB5*^+^ cancer cells may contribute to both tumor growth and structural remodeling within the tumor microenvironment. Survival analysis of the TCGA cervical cancer cohort revealed a borderline significant association between high *SERPINB5* expression and reduced overall survival (*p* = 0.062), suggesting a potential association between *SERPINB5* upregulation and adverse prognosis (Fig. 5G).

We next performed SERPINB5 knockdown to explore its downstream effects (Fig. 5H, I, and Table S8). Silencing of SERPINB5 led to broad suppression of oncogenic programs, including those related to stem cell population maintenance and epithelial to mesenchymal transition (Fig. 5J). Notably, several genes, such as *ITGA6*, *NOTCH3*, and *NOTCH2*, also showed elevated expression in *SERPINB5*^+^ cancer cells, suggesting potential stem-like features of this subpopulation [54–56] (Fig. S5). Enrichment analysis of genes downregulated upon SERPINB5 knockdown revealed suppression of oncogenic programs, including those involved in vasculature development, Wnt signaling, and cell morphogenesis, which are critical for tumor progression [57, 58] (Fig. 5K). Collectively, these findings identify *SERPINB5*^+^ cancer cells as a functionally distinct malignant subpopulation that promotes tumor progression by supporting niche architecture and enhancing proliferative capacity.

### SERPINB5 depletion suppresses tumor progression

**Fig. 6:**
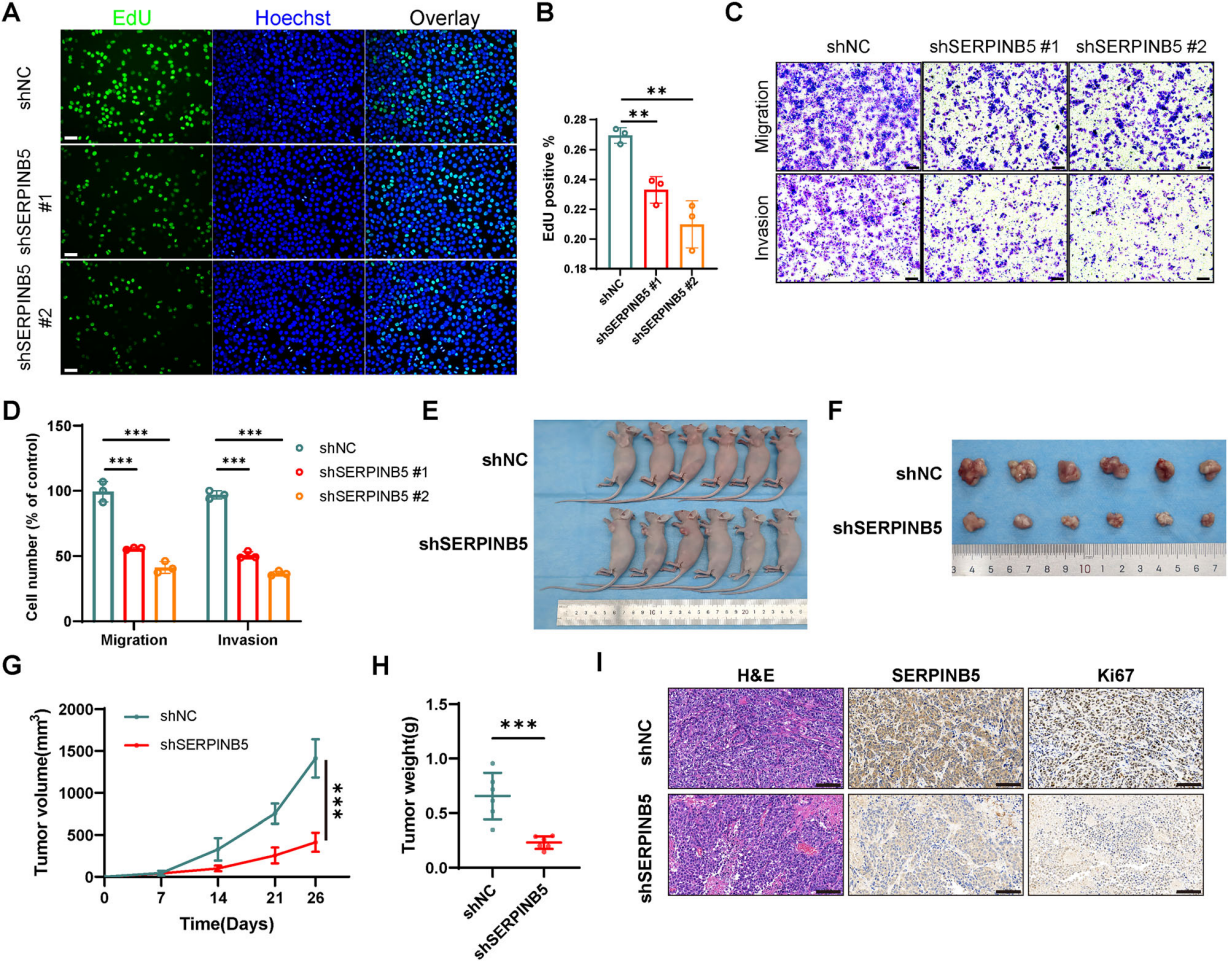
SERPINB5 promotes cervical cancer cell proliferation, metastasis, and tumor growth in vitro and in vivo. **A** Representative immunofluorescence images showing EdU (green) and Hoechst (blue) staining in HeLa cells transduced with shNC, shSERPINB5 #1, or shSERPINB5 #2. Scale bars, 100 μm. **B** Quantification of EdU-positive cells. Data are presented as mean ± SD from three independent experiments (*n* = 3). **C** Representative images of crystal violet-stained HeLa cells in transwell migration and invasion assays following SERPINB5 knockdown. Scale bars, 100 μm. **D** Quantification of migrated and invaded cells (*n* = 3). Data are presented as mean ± SD. **E** Representative images of nude mice bearing subcutaneous tumors derived from HeLa cells transduced with shNC or shSERPINB5 (*n* = 6 per group). **F** Photographs of tumors excised at the endpoint (*n* = 6 per group). **G** Tumor volumes were measured at the indicated time points. Data represent mean ± SD (*n* = 6). **H** Final tumor weights at sacrifice. Data represent mean ± SD (*n* = 6). **I** H&E staining and IHC for SERPINB5 and Ki67 were performed on tumor sections from xenograft mice. Scale bars, 100 μm. Statistical significance was determined using the two-tailed unpaired t-test (**B**, **D**, **H**) and two-way ANOVA followed by multiple comparisons test (**G**). NS not significant for *P* > 0.05, *P < 0.05, **P < 0.01, ***P < 0.001.

To functionally interrogate the role of *SERPINB5*^+^ cells in driving tumor aggressiveness, we silenced SERPINB5 in HeLa and SiHa cells and assessed multiple tumorigenic phenotypes. EdU assays showed significantly decreased proliferation after *SERPINB5* knockdown in both cell lines (Figs. 6A, B and S6A–D), supporting the notion that *SERPINB5*⁺ cells represent a highly proliferative malignant subpopulation. Transwell assays further demonstrated that loss of SERPINB5 significantly impaired cell migration and invasion in both models (Figs. 6C, D and S6E, F), suggesting that SERPINB5 prompts metastatic competence.

To validate the pro-tumorigenic role of SERPINB5 in *vivo*, we performed subcutaneous xenograft experiments in immunodeficient mice using SERPINB5-deficient and control HeLa cells. Female BALB/c nude mice transplanted with SERPINB5-knockdown cells developed visibly smaller tumors compared to controls (Fig. 6E, F). Notably, average tumor volume at endpoint decreased from 1414 mm^3^ in control mice to 413 mm^3^ in the knockdown group (Fig. 6G). Final tumor weights were also significantly reduced from 0.656 g to 0.231 g (Fig. 6H), indicating a sustained inhibitory effect on tumor growth. Moreover, immunohistochemical staining for Ki67 in xenograft tumors revealed a markedly lower proliferative index in the SERPINB5-depleted group compared to controls (Fig. 6I).

Together with the ST and scRNA-seq findings described above, these in vitro and in vivo experiments further support SERPINB5 as a functional oncogenic driver that promotes proliferative and metastatic phenotypes in cervical cancer.

### SERPINB5 modulates microtubule dynamics and the cell cycle to regulate microtubule-targeting chemotherapy response

**Fig. 7:**
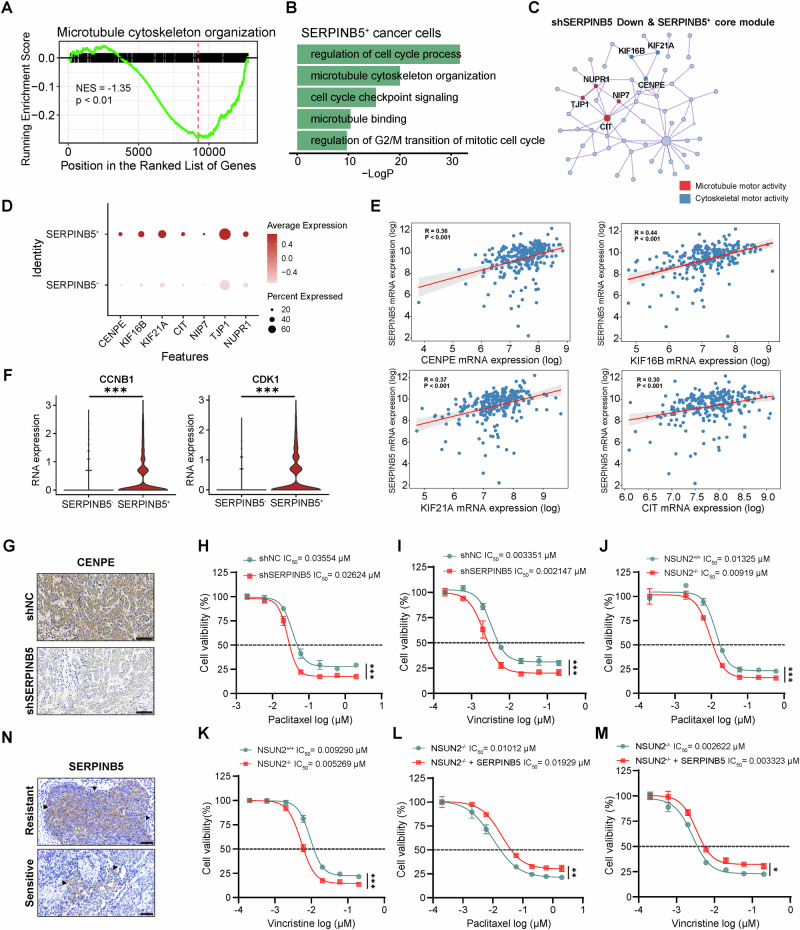
SERPINB5 modulates microtubule dynamics and cell cycle progression to regulate microtubule-targeting chemotherapy response in cervical cancer. **A** GSEA analysis of transcriptomic data from SERPINB5-knockdown HeLa cells revealed significant downregulation of pathways related to microtubule cytoskeleton organization. **B** Functional enrichment analysis comparing *SERPINB5*^+^ and *SERPINB5*^-^ cancer cells in scRNA-seq data. **C** PPI network visualization of the core module genes downregulated by shSERPINB5 and associated with *SERPINB5*⁺ cells. Nodes represent genes, and edges represent interactions. Colors indicate gene functions. **D** Dot plot showing the expression of selected genes in *SERPINB5*⁺ and *SERPINB5*^-^ cells. The size of the dots represents the percentage of expressing cells, and the color represents the average expression. **E** Correlation between *SERPINB5* mRNA expression and the expression of *CENPE*, *KIF16B, KIF21A* and *CIT* in TCGA cervical cancer dataset. **F** Violin plots showing the RNA expression of *CCNB1* and *CDK1* in *SERPINB5*⁺ and *SERPINB5*^-^ cancer cells (Wilcoxon rank-sum test). **G** Representative IHC staining of CENPE in xenograft tumor sections from HeLa cells transduced with shNC or shSERPINB5. Scale bars, 100 μm. HeLa cells transduced with shNC or shSERPINB5 were treated with paclitaxel (**H**) or vincristine (**I**) at gradient concentrations for 48 h, and both cell viability and IC_50_ values were measured using CCK-8 assay (*n*= 3). WT HeLa cells or NSUN2^–/–^ HeLa cells were treated with paclitaxel (**J**) or vincristine (**K**) for 48 h, and IC_50_ values were determined by CCK-8 assay (*n* = 3). NSUN2^–/–^ HeLa cells ectopically expressing an empty vector or *SERPINB5* were treated with paclitaxel (**L**) or vincristine (**M**) at gradient concentrations for 48 h, and cell viability as well as IC_50_ values were measured using CCK-8 assay (*n* = 3). **N** Representative IHC staining of SERPINB5 in tumor sections from paclitaxel-resistant and -sensitive tumors. Scale bars, 50 μm. Statistical significance was determined using two-way ANOVA followed by multiple comparisons test. Data are presented as mean ± SD from at least three independent experiments. NS not significant for *P* > 0.05, *P < 0.05, **P < 0.01, ***P < 0.001.

Having identified SERPINB5 as a pro-tumorigenic driver in cervical cancer, we next investigated its potential role in mediating chemoresistance. Transcriptomic profiling of SERPINB5 knockdown HeLa cells revealed significant downregulation of pathways related to microtubule cytoskeleton organization (Fig. 7A). Consistently, scRNA-seq analysis comparing *SERPINB5*^+^ versus *SERPINB5*^-^ cancer cells revealed strong enrichment of gene signatures involved in cell cycle regulation, G2/M transition, checkpoint control, and microtubule binding in *SERPINB5*^+^ cancer cells (Fig. 7B). To define downstream effectors, we constructed a PPI network from genes downregulated upon SERPINB5 knockdown. This analysis revealed two highly interconnected functional modules: one enriched for microtubule motor proteins (e.g., *CENPE*, *KIF16B*, *KIF21A*) and the other for cytoskeletal motor components (e.g., *CIT*, *TJP1*) (Fig. 7C). These genes were preferentially expressed in *SERPINB5*^+^ cancer cells and exhibited strong positive correlation with *SERPINB5* expression in TCGA cervical cancer datasets (Figs. 7D, E and S7A). Overexpression of SERPINB5 led to significant upregulation of these genes (Fig. S7B–E). In parallel, canonical mitotic regulators such as *CCNB1* and *CDK1* were also highly expressed in *SERPINB5*^+^ cancer cells, further supporting a broad role of *SERPINB5* in orchestrating both microtubule dynamics and cell cycle progression [59–62] (Fig. 7F). Among them, CENPE, a mitotic kinesin essential for spindle attachment and chromosomal alignment, emerged as a key hub [63]. Notably, immunohistochemical analysis of xenograft tumors with SERPINB5 knockdown showed reduced CENPE expression (Fig. 7G).

These findings suggest that SERPINB5 activates a mitotic gene program that functionally antagonizes the cytotoxic mechanism of microtubule-targeting agents. Given that paclitaxel and vincristine exert their anti-tumor effects by perturbing microtubule dynamics and inducing mitotic arrest that leads to cell death [7, 64], we next assessed whether SERPINB5 modulates cellular resistance to these agents. Functional assays revealed that SERPINB5 knockdown in HeLa cells significantly reduced the IC_50_ values for paclitaxel (0.03554 μM to 0.02624 μM) and vincristine (0.003351 μM to 0.002147 μM), indicating restored sensitivity to microtubule-targeting agents (Fig. 7H, I). Importantly, replication of these experiments in SiHa cells confirmed that *SERPINB5* knockdown robustly increased sensitivity to both paclitaxel and vincristine (Fig. S8A, B), strengthening the generalizability of our findings. Similarly, NSUN2 knockout produced comparable effects (Figs. 7J, K and S8C, D). Most strikingly, ectopic expression of SERPINB5 in NSUN2^–/–^ HeLa cells and NSUN2^–/–^ SiHa cells partially restored resistance to paclitaxel and vincristine, demonstrating that SERPINB5 is a key effector of NSUN2-driven epitranscriptomic regulation of chemoresistance (Figs. 7L, M and S8E, F). We also examined cisplatin resistance and found that *SERPINB5* knockdown had a minimal impact, indicating this regulatory axis specifically modulates resistance to microtubule-targeting agents (paclitaxel and vincristine) (Fig. S8G, H). Immunohistochemical staining showed markedly elevated SERPINB5 protein levels in paclitaxel-resistant tumors compared to sensitive ones (Fig. 7N).

**Fig. 8:**
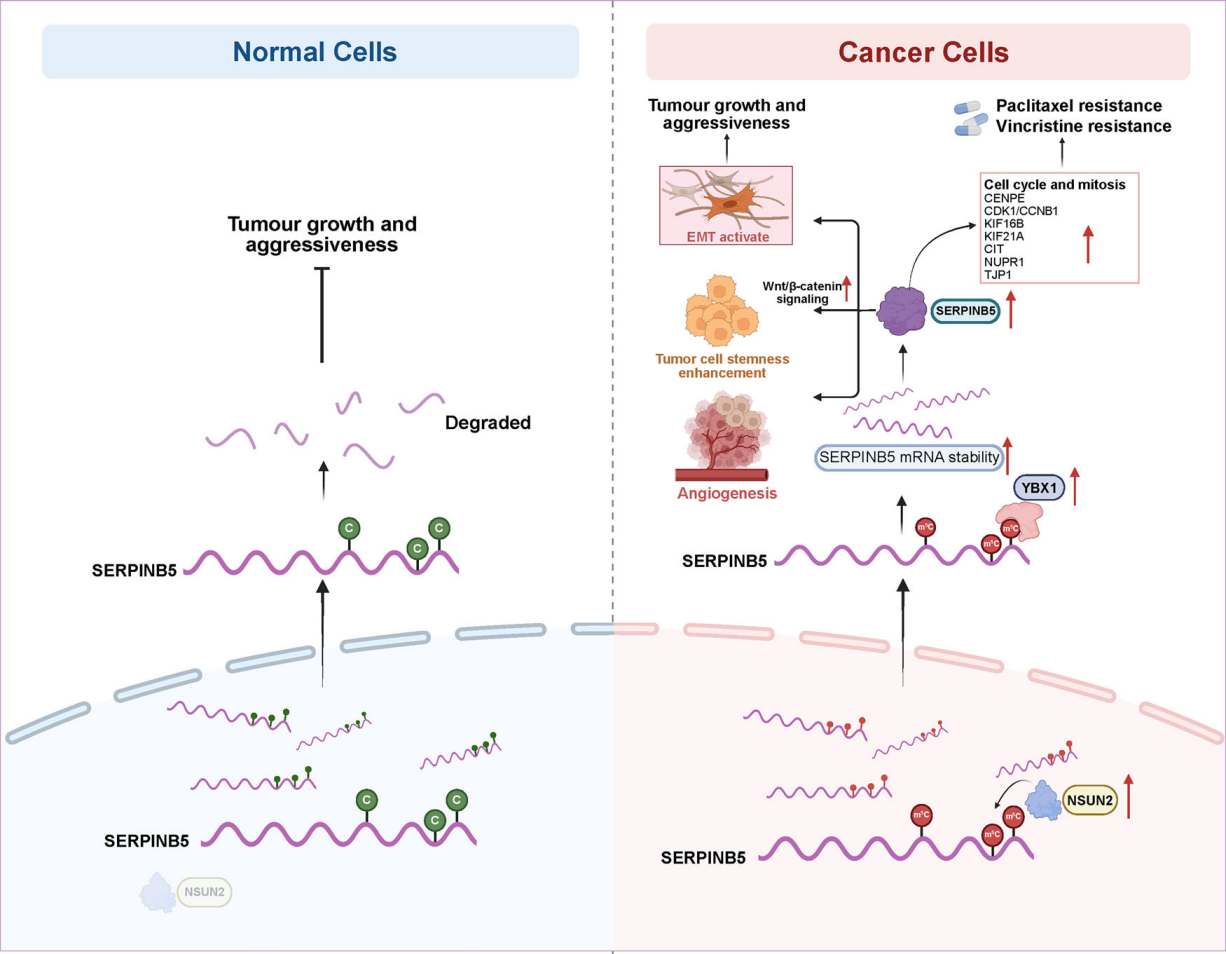
Schematic model of the NSUN2/YBX1–SERPINB5 axis in normal and cancer cells. This model illustrates how SERPINB5, initially expressed at low levels in normal cells, becomes a key effector of malignancy in cervical cancer through regulation by the NSUN2–SERPINB5 axis. In normal epithelial cells (left panel), low SERPINB5 expression limits its oncogenic potential. In cancer cells (right panel), NSUN2-mediated m^5^C methylation and YBX1 binding enhance *SERPINB5* mRNA stability. The upregulated SERPINB5 promotes multiple oncogenic processes, including cancer stemness, angiogenesis, and EMT. In addition, SERPINB5 upregulates mitotic regulators such as CDK1, CCNB1, and KIF16B, promoting tumor cell proliferation and resistance to paclitaxel- and vincristine-induced mitotic arrest. Illustration created with BioRender (https://biorender.com/).

Together, these results delineate a hitherto undefined epitranscriptomic mechanism of chemoresistance: NSUN2-mediated m^5^C deposition stabilizes *SERPINB5* mRNA, leading to elevated protein expression. In turn, SERPINB5 activates a mitosis-centric transcriptional program involving microtubule motor proteins (e.g., CENPE, KIFs) and cell cycle drivers (e.g., CDK1, CCNB1), ultimately blunting the mitotic arrest induced by microtubule-targeting agents like paclitaxel and vincristine (Fig. 8). This NSUN2–SERPINB5 axis not only establishes a novel regulatory link between RNA methylation and microtubule-inhibitor resistance but also highlights SERPINB5 as a promising biomarker and therapeutic target for improving chemotherapeutic response in cervical cancer.

## Discussion

Our study uncovers a novel epitranscriptomic mechanism in cervical cancer, whereby NSUN2-mediated m^5^C RNA methylation stabilizes *SERPINB5* and reinforces mitotic transcriptional programs. Rather than merely promoting tumor progression, this axis may also contribute to selective tolerance of microtubule-targeting chemotherapeutics, highlighting a previously unexplored link between RNA methylation and cell-cycle–associated drug evasion. These findings provide mechanistic insight into chemotherapy failure in cervical and potentially other epithelial malignancies (Fig. 8).

By generating the first base-resolution m^5^C methylome of cervical cancer, we observed widespread m^5^C hypermethylation of transcripts involved in focal adhesion and adherens junction pathways, which transmit mechanical and biochemical signals from the membrane to the nucleus, potentially influencing chromatin architecture, transcriptional regulation, and cytoskeletal dynamics. These observations suggest that m^5^C methylation may help coordinate extracellular cues with nuclear programs, forming an integrated network that supports tumor progression [42]. This concept is supported by growing evidence across cancer types [65–68]. To identify functional m^5^C effectors, we developed MORGAN, a modeling framework that integrates methylation intensity, transcriptional output, and cancer-specific remodeling. This approach identified SERPINB5 as a key candidate. SERPINB5 has previously been described as a regulator with both tumor-suppressive and oncogenic roles across different malignancies [31, 34, 69, 70]. Our data clearly support a pro-oncogenic role for SERPINB5 in cervical cancer. Mechanistically, NSUN2-mediated m^5^C deposition facilitates YBX1 binding and stabilizes *SERPINB5* mRNA.

Although NSUN2 knockout reduced SERPINB5 expression by approximately 50%, the incomplete loss of SERPINB5 suggests that additional mechanisms may contribute to its post-transcriptional stability. First, *SERPINB5* transcript stability may also be maintained by alternative RNA-binding proteins, such as HuR (ELAVL1) or IGF2BP family members, which promote mRNA stabilization [71, 72]. Second, given that m^5^C modifications can be partially redundant among RNA methyltransferases, other enzymes (e.g., NSUN6 or TRDMT1) may contribute to basal m^5^C deposition on *SERPINB5* transcripts in the absence of NSUN2 [16, 73]. Finally, feedback regulation via transcriptional activation may also sustain SERPINB5 expression to some degree, as it is often co-regulated with proliferative and stress-response programs [74]. These findings collectively suggest that while NSUN2-driven m^5^C methylation is a major determinant of *SERPINB5* stability, multiple parallel mechanisms likely cooperate to sustain its oncogenic expression in cervical cancer.

*SERPINB5*⁺ cancer cells define a transcriptionally distinct subpopulation enriched in mitotic and cytoskeletal programs, which may contribute to sustained proliferation and tumor microenvironment remodeling. Notably, SERPINB5 appears to protect cells from the cytotoxic effects of microtubule-targeting agents such as paclitaxel and vincristine, which disrupt microtubule dynamics, induce prolonged mitotic arrest, and ultimately trigger cell death [8, 75–77]. Critically, SERPINB5 overexpression enables cancer cells to evade this fatal arrest. It achieves this by upregulating key mitotic effectors, including the mitotic kinesins (e.g., CENPE) and G2/M regulators (e.g., CDK1, CCNB1) [78]. Interestingly, this effect was selective for microtubule-targeting agents, with minimal impact observed on cisplatin response. This specificity underscores that the NSUN2–SERPINB5 axis and its downstream mitotic/microtubule motor program counteract the cytotoxic mechanisms of paclitaxel and vincristine, rather than conferring non-specific resistance [79].

SERPINB5 expression could potentially serve as a predictive biomarker for microtubule-targeting drug response. Elevated SERPINB5 levels were observed in tumors classified as paclitaxel-resistant, highlighting its potential utility in stratifying patients prior to treatment and guiding therapeutic decisions. This is particularly important given the widespread use of paclitaxel in cervical cancer, especially in low- and middle-income regions where alternative therapies are limited, and resistance remains a major challenge [80]. Unlike classical resistance mechanisms such as ABC transporter-mediated drug efflux [81], SERPINB5-driven resistance operates by enhancing mitotic progression and potentially microtubule stability under chemotherapeutic stress, suggesting mechanistic complementarity. Co-targeting these pathways alongside conventional resistance mechanisms may offer a rational strategy to enhance drug efficacy. Notably, these conclusions remain preliminary and warrant further validation.

Several limitations warrant consideration. Although we profiled m^5^C modifications and transcriptomes in clinical tumor specimens, functional experiments were primarily conducted in cell lines and xenograft models. Future studies using patient-derived organoids or genetically engineered mouse models will be essential to validate tissue-specific dependencies and therapeutic efficacy in a more physiologically relevant setting. Moreover, although our data highlight the therapeutic potential of targeting components of the NSUN2/YBX1–SERPINB5 axis, including NSUN2 itself, YBX1-mediated RNA binding, and SERPINB5 effectors, the development of selective small-molecule inhibitors against these targets remains in its infancy [82]. Advancing our understanding of the structural interfaces within this axis may accelerate future drug discovery.

In conclusion, our study reveals a novel m^5^C-dependent mechanism linking NSUN2-mediated RNA methylation to *SERPINB5* stabilization and tumor progression, which may contribute to selective tolerance to microtubule-targeting chemotherapeutic agents, such as paclitaxel and vincristine, in cervical cancer. These findings broaden our understanding of epitranscriptomic remodeling in solid tumors and provide a rationale for targeting the NSUN2–SERPINB5 axis to improve therapeutic outcomes.

## Materials and methods

### RNA-seq library preparation and sequencing

Total RNA was extracted from tumor and normal samples using TRIzol reagent (Invitrogen). Genomic DNA contamination was removed by DNase I digestion. RNA purity was assessed by measuring the A260/A280 ratio. RNA integrity was evaluated via 1.5% agarose gel electrophoresis. A total of 2 µg of RNA per sample was used for stranded RNA-seq library preparation. Ribosomal RNA (rRNA) was depleted using the Ribo-off rRNA Depletion Kit (Human/Mouse/Rat) (Illumina, MRZG12324), followed by library construction using the KC-Digital Stranded mRNA Library Prep Kit for Illumina (Wuhan Seqhealth, DR08502), according to the manufacturer’s instructions. This kit incorporates unique molecular identifiers (UMIs) consisting of 8 random nucleotides to label pre-amplified cDNA molecules, thereby minimizing PCR and sequencing duplication bias. Library fragments ranging from 200 to 500 bp were size-selected, quantified, and sequenced using the DNBSEQ-T7 platform with paired-end 150 bp (PE150) reads.

### RNA-seq analysis

RNA-seq reads were aligned to the human genome (hg38) using STAR (v2.7.11b) [83]. Gene expression levels were quantified using the featureCounts function from the SubReads package (v2.0.6) [84]. Differentially expressed genes (DEGs) were identified using the DESeq2 package (v1.42.0) [85], with the following thresholds: Benjamini-Hochberg adjusted *p*-value < 0.01 and fold change >2. Functional enrichment analysis was conducted using the clusterProfiler package (v4.10.0) [86] to evaluate the enrichment of DEGs in specific biological pathways. Additionally, pathway and functional enrichment analyses, as well as protein-protein interaction (PPI) module analysis, were performed using Metascape (http://metascape.org).

The bulk RNA-seq data of cancer samples were downloaded from the TCGA CESC cohort (https://portal.gdc.cancer.gov/projects/TCGA-CESC) using the gdc-client tool. A total of 257 expression files and 255 clinical files were available for analysis. To ensure the robustness of our analysis, low-expressed genes, defined as those with counts equal to zero in more than 25% of the samples, were filtered out. After filtering, data from 233 cancer patients were included in the study. Gene expression correlations were calculated using the np.corrcoef() function from the NumPy module in Python. Additionally, the significance of the correlations was assessed using the stats.pearsonr() function from the Stats module. The expression profile of *SERPINB5* across various cancer types and its association with overall survival in cervical cancer patients were analyzed using GEPIA (http://gepia.cancer-pku.cn/detail.php). For additional validation of *NSUN2* mRNA expression, two cervical cancer bulk RNA-seq datasets were retrieved from the Gene Expression Omnibus (GEO) under accession numbers GSE63514 and GSE138080 [87, 88].

### BS-seq library preparation and sequencing

Total RNA used for BS-seq was the same as described in Section 1.1. For each sample, 1 μg of rRNA-depleted RNA was mixed with 5 ng of in vitro-transcribed luciferase RNA as an internal control and treated with the EZ RNA Methylation Kit (Zymo Research) according to the manufacturer’s instructions. Specifically, 20 μL RNA (including spike-in) was mixed with 130 μL RNA conversion reagent in a 200 μL RNase-free PCR tube, briefly centrifuged, and incubated in a thermal cycler for bisulfite conversion using the following cycling program: 70 °C for 5 min and 64 °C for 45 min, repeated for three cycles, and held at 4 °C.

After conversion, the RNA was purified using the spin-column protocol provided by the kit. The column was equilibrated with 250 μL RNA binding buffer before sample loading. Ethanol (95–100%) was added to the sample, and the mixture was applied to the column. After washing with RNA wash buffer and desulfonation with RNA desulfonation buffer at room temperature for 30 min, additional washes were performed to remove residual salts. The RNA was eluted with 12 μL of pre-warmed (60 °C) RNase-free water and stored at –80 °C. The library construction and sequencing were performed following the same procedure as described in Section 4.2.

### BS-seq data analysis

The BS-seq data were aligned to the human reference genome (GRCh38) using meRanGs, a module within the meRanTK suite (v1.3.0), with default parameters to enable bisulfite-converted read mapping [89]. Methylation calling was performed using meRanCall to quantify methylation levels at cytosine sites, applying a filtering threshold of FDR < 0.01 and minimum coverage ≥10×. Differential methylation analysis between normal and cancer groups was conducted using meRanCompare, with size factor normalization performed via the estimateSizeFactors.pl script and significance determined by an FDR < 0.05. Methylation sites were annotated to genomic features using the Ensembl GTF file (release 102) via meRanAnnotate. Motif enrichment analysis was conducted using HOMER (v4.11) [90] and visualized with the ggseqlogo R package (v0.1) [91]. Methylation genomic region proportions were annotated using ChIPseeker (v1.38.0) [92].

### Single-cell RNA-seq data processing

Single-cell RNA-seq data were processed following the same pipeline as previously described [52]. Briefly, reads were aligned to the GRCh38 human reference genome using Cell Ranger (v7.0.1). Cells were filtered using stringent QC thresholds in Seurat (v5.0.1) [93], including detected genes (200–10,000), UMI count (<60,000), mitochondrial content (<25%), erythrocyte gene expression (<25%), and ribosomal gene fraction (<5%). Data integration was performed using Harmony (v1.1.0) [94], and clustering was conducted with PCA, UMAP, and graph-based clustering (resolution = 0.8). Cell types were annotated with canonical markers. Differential expression analyses were independently conducted between *SERPINB5*^+^ and *SERPINB5*^–^ cancer cells, *NSUN2*^+^ and *NSUN2*^-^ cells, as well as *YBX1*^+^ and *YBX1*^-^ cell populations using the Seurat’s FindAllMarkers function with adjusted *P* < 0.05.

### Stereo-seq data analysis

Stereo-seq data were processed and analyzed following the same procedure as previously described [52]. The dataset used in this study was obtained from the Genome Sequence Archive at the BIG Data Center (accession number: HRA007417). To systematically characterize the spatial transcriptomic landscape of tumor regions, this study employed a spatial binning grid of 50 × 50 (250 × 250 μm) as the fundamental analytical unit (spot). Leveraging cell-type signatures defined by single-cell RNA sequencing (scRNA-seq), we applied the cell2location spatial deconvolution model (v0.5-alpha) to map Stereo-seq data, with key parameters set to N_cells_per_location = 6 and detection_alpha = 20 [95].

### Computational model: MORGAN

To systematically identify genes driven by RNA m^5^C methylation and implicated in cancer development, we developed a computational model named MORGAN (*M*^*5*^*C-Oriented RNA Gene Identification via Adaptive Gaussian Mixture Network*). This model integrates m^5^C methylation profiles from RNA BS-seq with corresponding gene expression data from RNA-seq. During preprocessing, RNA-seq data were normalized using CPM (Counts Per Million) and strictly matched to m^5^C data by gene names. Genes with low expression (those with zero counts in more than 25% of samples) were filtered out. Subsequently, Pearson’s correlation coefficient (r) was used to analyze the correlation between gene methylation levels and gene expression. Genes with strong correlations (|r|>0.6) were selected to form a candidate gene list.

To address the m^5^C heterogeneity of candidate genes, a Gaussian Mixture Model combined with the R package mclust (v6.0.1) was employed for clustering analysis. The model set the clustering number range to 1-3 (G = 1–3), evaluated the optimal number of clusters using the Bayesian Information Criterion (BIC), and classified samples into high-, medium-, and low-methylation states. The m^5^C methylation state of each gene was assigned to specific categories through Maximum A Posteriori probability to ensure biologically plausible cluster assignment.

To quantify the methylation difference between cancer and normal samples and estimate the potential driver effect, a metric called the Differential Methylation (DM) value was defined. This value was calculated through a two-step weighted strategy. First, the difference in mean methylation between cancer and normal samples was computed for each methylation state. Second, these differences were adjusted by a custom weighting algorithm (Eq. 1) that incorporates both the state complexity (${O}_{i}$) and the cluster-specific methylation means (${C}_{i}$).

1 $${DM}-{value}\,=\frac{\,{O}_{i}\,\cdot \,{C}_{i}\,\cdot \,{\sum }_{i}\log \left({\mu }_{{cancer},i}\,-{\mu }_{{normal},i}\,+\,1\right)}{{\sum }_{i}{O}_{i\,}\cdot \,{C}_{i}\,}$$

Here, ${\mu }_{{cancer},i}$ and $\mu \_({normal},i)$ are the mean methylation levels for cancer and normal samples, respectively, in methylation state $i$; ${O}_{i}$ reflects the complexity (entropy) of state $i$; and ${C}_{i}$ reflects the number of samples assigned to state $i$. The logarithmic function enhances the contribution of hypermethylated states, while the complexity and weight terms adjust for heterogeneity and prevalence across states. A higher DM value indicates that a gene exhibits a stronger m^5^C-driven effect associated with cancer.

### Cells and reagents

HEK293T (CCTCC, Cat# GDC0187, RRID: CVCL_0063), HeLa (CCTCC, Cat# GDC0009, RRID: CVCL_0030), and SiHa (CCTCC, Cat# GDC0110, RRID: CVCL_0032) cells were obtained from the China Center for Type Culture Collection (CCTCC). All cell lines were authenticated by short tandem repeat (STR) profiling and confirmed to be free of mycoplasma contamination by the supplier. All cells were cultured in Dulbecco’s Modified Eagle Medium (DMEM) supplemented with 10% fetal bovine serum (FBS), 100 U/mL penicillin, and 100 µg/mL streptomycin at 37 °C in a humidified incubator with 5% CO_2_. Plasmid transfections were performed using Lipofectamine 3000 (Invitrogen, L3000015) or Neofect (Neofect, TF201201) according to the manufacturer’s protocols. Small interfering RNAs (siRNAs) were transfected using Lipofectamine RNAiMAX (Invitrogen, 13778150) following the manufacturer’s instructions. Actinomycin D was purchased from MedChemExpress (MCE, HY-17559). Paclitaxel, vincristine, and cisplatin were purchased from Shanghai Yuanye Biological Technology Co., Ltd.

### Immunohistochemical staining

Paraffin-embedded tumor or normal sections (4 μm thick) were deparaffinized, rehydrated, and subjected to antigen retrieval, followed by blocking of endogenous peroxidase activity with 3% hydrogen peroxide. The sections were then blocked with 3% bovine serum albumin for 30 min at room temperature and incubated overnight at 4 °C with primary antibodies. The primary antibodies used were: rabbit anti-NSUN2 (Proteintech, 20854-1-AP), rabbit anti-YBX1 (Proteintech, 20339-1-AP), rabbit anti-SERPINB5 (Proteintech, 11722-1-AP), rabbit anti-CENPE (Proteintech, 28142-1-AP), and rabbit anti-Ki-67 (Huilan Biotech, ABB00008). After PBS washes, sections were incubated with HRP-conjugated, species-specific secondary antibodies (goat anti-rabbit, Abcam, ab205718) for 50 min at room temperature. Following additional PBS washes, a freshly prepared DAB solution was applied for color development and stopped with ddH_2_O. Nuclei were counterstained with Harris hematoxylin. After dehydration and mounting with neutral resin, sections were imaged under a light microscope for analysis.

### Multiplex immunofluorescence assay

Tumor or normal tissues were sectioned at a 4 μm thickness. After deparaffinization and rehydration, antigen retrieval was performed by boiling the slides in citric acid buffer (pH 6.0) for 2 min. Endogenous peroxidase activity was blocked using 3% hydrogen peroxide for 20 min, followed by protein blocking with 10% goat serum at 37 °C for 30 min.

Multiplex staining was conducted using a cyclic immunofluorescence protocol. Each cycle included sequential incubation with primary and HRP-conjugated secondary antibodies, followed by tyramide signal amplification (TSA). The TSA-antibody complex was removed via microwave treatment in citric acid buffer (pH 6.0) at 97 °C for 5 min, allowing subsequent rounds of staining. Each staining cycle included repeated antigen retrieval and protein blocking steps as described.

After completion of all staining cycles, nuclei were counterstained with DAPI (Servicebio, G1012-10ML) for 5 min. Slides were scanned using the Pannoramic MIDI system (3DHISTECH, Budapest).

Primary antibodies used included: rabbit-NSUN2 (Proteintech, 20854-1-AP), rabbit-YBX1 (Proteintech, 20339-1-AP), and rabbit-SERPINB5 (Proteintech, 11722-1-AP). HRP-labeled goat anti-rabbit secondary antibodies (Abcam, ab205718) were applied for 1 h at 37 °C.

### RNA isolation and real-time quantitative PCR (qPCR)

Total RNA was isolated from cultured cells or tumor tissues using TRIzol reagent (Invitrogen, 15596018), and reverse-transcribed into cDNA using PrimeScript RT Reagent Kit (Takara, RR037A). Real-time quantitative PCR was carried out through the ABI 7500 Real Time PCR System by SYBR Green Master Mix (YEASEN, 11199ES03) to assess gene expression. Each experiment was independently repeated three times. GAPDH was used as the internal control, and relative mRNA levels were calculated using the ΔΔCt method. Primer sequences are provided in Supplementary Table S9.

### mRNA stability assay

To assess mRNA half-life, cells were seeded in 24-well plates and transfected for 36 h. The culture medium was then replaced with fresh medium containing actinomycin D (2 μg/mL), and cells were incubated at 37 °C. At the indicated time points (0, 3, 6, and 9 h), cells were harvested by adding 500 μL of TRIzol reagent per well, followed by transfer of the lysate to 1.5 mL microcentrifuge tubes. The wells were rinsed once with PBS to remove residual TRIzol. Total RNA was extracted from each time point, reverse transcribed into cDNA, and analyzed by qPCR.

### RNA-binding protein immunoprecipitation qPCR (RIP-qPCR)

A total of 200 μg RNA was incubated with anti-YBX1 antibody in 800 µL of IPP buffer (150 mM NaCl, 0.1% NP-40, 10 mM Tris-HCl, pH 7.4) for 2 h at 4 °C. Subsequently, 30 µL of protein A/G beads were added, and the mixture was incubated overnight. The beads were washed five times with IPP buffer before RNA extraction and qPCR analysis.

### EdU cell proliferation assay

Cell proliferation was assessed using the EdU Cell Proliferation Assay Kit (Abbkine, KTA2030) following the manufacturer’s instructions. Cells were seeded into 96-well plates at a density of 1.5 × 10^5^ cells/mL and incubated with 10 μM EdU for 2 h. After incubation, the culture medium was removed, and the cells were fixed with 4% paraformaldehyde for 15 min at room temperature, washed with PBS, and permeabilized with 0.5% Triton X-100 for 20 min. Subsequently, 200 μL of Click-iT reaction mixture was added to each well and incubated for 30 min in the dark. EdU-positive cells were visualized under a Leica fluorescence microscope.

### Transwell migration and invasion assay

Migration and invasion assays were performed using an 8 μm pore size transwell system (Corning, 3422). For the migration assay, HeLa cells stably expressing shSERPINB5 or control shRNA were seeded into the upper chamber (5 × 10^4^ cells) in serum-free medium. For the invasion assay, the upper chamber was pre-coated with Matrigel (Corning), and the subsequent steps were similar to those in the migration assay. Medium containing 30% FBS was added to the lower chamber as a chemoattractant. After incubation for 36 h, migrated or invaded cells on the lower surface of the membrane were fixed with 4% methanol-free formaldehyde (Hushi, 10010018) and stained with 0.1% crystal violet (Macklin, C805209). Five random fields per insert were imaged under a 20× microscope, and cell numbers were quantified using ImageJ. Migration and invasion indices were calculated by normalizing to the control group.

### Chemotherapy drug sensitivity assay

Paclitaxel and cisplatin stock solutions were prepared according to the manufacturer’s instructions. Cells were seeded into 96-well plates with culture medium containing 10% FBS and allowed to attach overnight. Subsequently, cells were treated with a series of concentrations of each drug. After 48 h of incubation, cell viability was measured using the Cell Counting Kit-8 (CCK-8, Vazyme, A311-01-AA) according to the manufacturer’s protocol. Briefly, a working solution was prepared by mixing 10 μL of CCK-8 reagent with 90 μL of culture medium. Then, 100 μL of this working solution was added to each well and incubated for 45 min at 37 °C. Absorbance at 450 nm was recorded using a microplate reader (Molecular Devices). Cell viability was calculated based on the absorbance values, and the half-maximal inhibitory concentration (IC_50_) was determined from dose-response curves using GraphPad Prism software.

### Plasmids and RNA interference

NSUN2 and SERPINB5 were individually cloned into the pCAGGS expression vector for overexpression studies. A catalytically inactive NSUN2 double mutant (I302A/C321A), previously reported to abolish m^5^C methyltransferase function [96, 97], was generated and cloned into the same vector. For gene silencing, both siRNAs and shRNAs were employed. The sequences of the siRNAs are listed in Supplementary Table S9, and knockdown efficiency was evaluated 36–48 h after transfection using immunoblot analysis or qPCR. For stable knockdown, gene-specific shRNA oligonucleotides were designed and cloned into the pLKO.1 vector (Addgene). Lentiviral particles were generated by co-transfecting HEK293T cells with the shRNA plasmid, psPAX2 packaging plasmid, and pMD2.G envelope plasmid. Viral supernatants were collected at 48 h, filtered, and used to infect target cells, followed by puromycin (Sangon Biotech, A606719) (2 µg/mL) selection to establish stable knockdown lines.

### Construction of NSUN2-knockout cell lines

Two sgRNAs targeting NSUN2 were cloned into the lentiCRISPR-v2 vector (Addgene) (sequences listed in Supplementary Table S9). HEK293T cells were co-transfected with lentiCRISPR-v2, psPAX2, and pMD2.G using Neofect reagent in six-well plates. After 48 h, lentiviral supernatants were collected, filtered (0.45 μm), and used to infect target cells in the presence of polybrene (8 μg/mL). To enhance transduction efficiency, cells were infected twice, followed by puromycin selection. Single-cell clones were obtained by limiting dilution in 96-well plates, expanded, and validated by immunoblot analysis and genomic PCR followed by sequencing.

### Immunoblot analysis

Cells were washed with PBS and lysed in NP-40 lysis buffer (50 mM Tris-HCl, pH 7.4, 150 mM NaCl, 1% NP-40) at 4 °C for 30 min, followed by boiling in 1% SDS for 10 min. Protein samples were mixed with SDS loading buffer, resolved by SDS-PAGE, and transferred onto nitrocellulose membranes (BioTrace). Membranes were blocked in TBS containing 0.1% Tween-20 (TBST) containing 5% non-fat milk or bovine serum albumin (BSA), incubated with primary antibodies, and then with HRP-conjugated secondary antibodies. Signals were detected using chemiluminescent substrate (Millipore, WBKLS0500) and visualized with X-ray film or a ChemiDoc Imaging System (Bio-Rad, 12003154).

### Reporter gene assays

Cells were seeded onto 24-well plates and transfected with various plasmid combinations. For overexpression experiments, cells were transfected with 50–100 ng of firefly luciferase reporter plasmid along with 20 ng of Renilla luciferase plasmid (pRL-TK) as an internal control to normalize transfection efficiency. For knockdown assays, siRNAs were first transfected, and 36 h later, the luciferase reporter and Renilla plasmids were introduced. Luciferase activities were measured sequentially using the Dual Luciferase Reporter Assay Kit (Vazyme, DL101) following the manufacturer’s instructions. Relative luciferase activity was calculated by normalizing firefly luciferase activity to that of Renilla luciferase. Each experiment was performed with at least three biological replicates.

### Tumor xenograft model

Female BALB/c nude mice (18–22 g, 4–5 weeks old) were housed under specific pathogen-free (SPF) conditions and acclimated for one week before experimentation. Mice were randomly divided into two groups (*n* = 6 per group): shNC and shSERPINB5. The sample size was chosen based on effect sizes observed in our pilot studies and is consistent with sample sizes commonly used in similar xenograft studies in the literature to ensure robust and reproducible results. Stable SERPINB5 knockdown in HeLa cells was confirmed by immunoblot analysis, while control cells showed no change in expression. Cells were trypsinized, washed twice with DMEM, and resuspended in ice-cold DMEM at 5 × 10^6^ cells per 150 μL. Cell suspensions were kept on ice and subcutaneously injected into the left forelimb region of each mouse.

Tumor growth was monitored at least twice weekly starting from day 7 post-inoculation by measuring tumor length (L) and width (W) with calipers. Tumor volume (V) was calculated using the formula: V = 0.5 × L × W^2^ (mm^3^). The animal experiments were performed by certified staff at the Center for Animal Experiments of Wuhan University and approved by the Institutional Animal Care and Use Committee (AUP # WP20250061). According to the approved protocol, mice were euthanized when tumor volume approached 2000 mm^3^ or at the end of the study period. In this study, the maximal tumor size was not exceeded in any mice. Due to the evident phenotypic differences (e.g., tumor size) between shNC and shSERPINB5 groups, blinding of the investigators during the experiment and outcome assessment was not feasible.

### Statistical analysis

All statistical analyses were performed using GraphPad Prism 9.0 software or as indicated in the respective method sections. All experiments were performed with at least three independent biological replicates, and the exact sample size (*n*) for each experiment is provided in the corresponding figure legends. Data are presented as follows: box plots display the median and the range from minimum to maximum values, while bar and line graphs show the mean ± SD. Details of data presentation and statistical methods are described in the figure legends. A P-value of less than 0.05 was considered statistically significant (*P < 0.05, **P < 0.01, ***P < 0.001).

## Supplementary information

Supplementary figures

Supplementary table

Original Western blots

## Data Availability

All analyses were conducted using publicly available software, as detailed in the Methods. The source code for the analysis is available at: https://github.com/chenyulab4126/RNA-m5C-project-of-cervical-cancer. The RNA BS-seq and RNA-seq raw sequencing data from this study have been deposited in the Genome Sequence Archive in BIG Data Center (https://bigd.big.ac.cn/), Beijing Institute of Genomics (BIG), Chinese Academy of Sciences, under the accession numbers: HRA012262 and HRA012263.
