# Supplementary material for: M5C-driven stabilization of SERPINB5 promotes cervical cancer progression and chemotherapy resistance
Source: Cell Death Dis. 2026 Feb 11;17(1):215. doi: 10.1038/s41419-026-08453-2 (PMC12921336; doi:10.1038/s41419-026-08453-2)
Supplement: Supplementary file 1 — Supplementary figures [file 41419_2026_8453_MOESM1_ESM.docx]

**
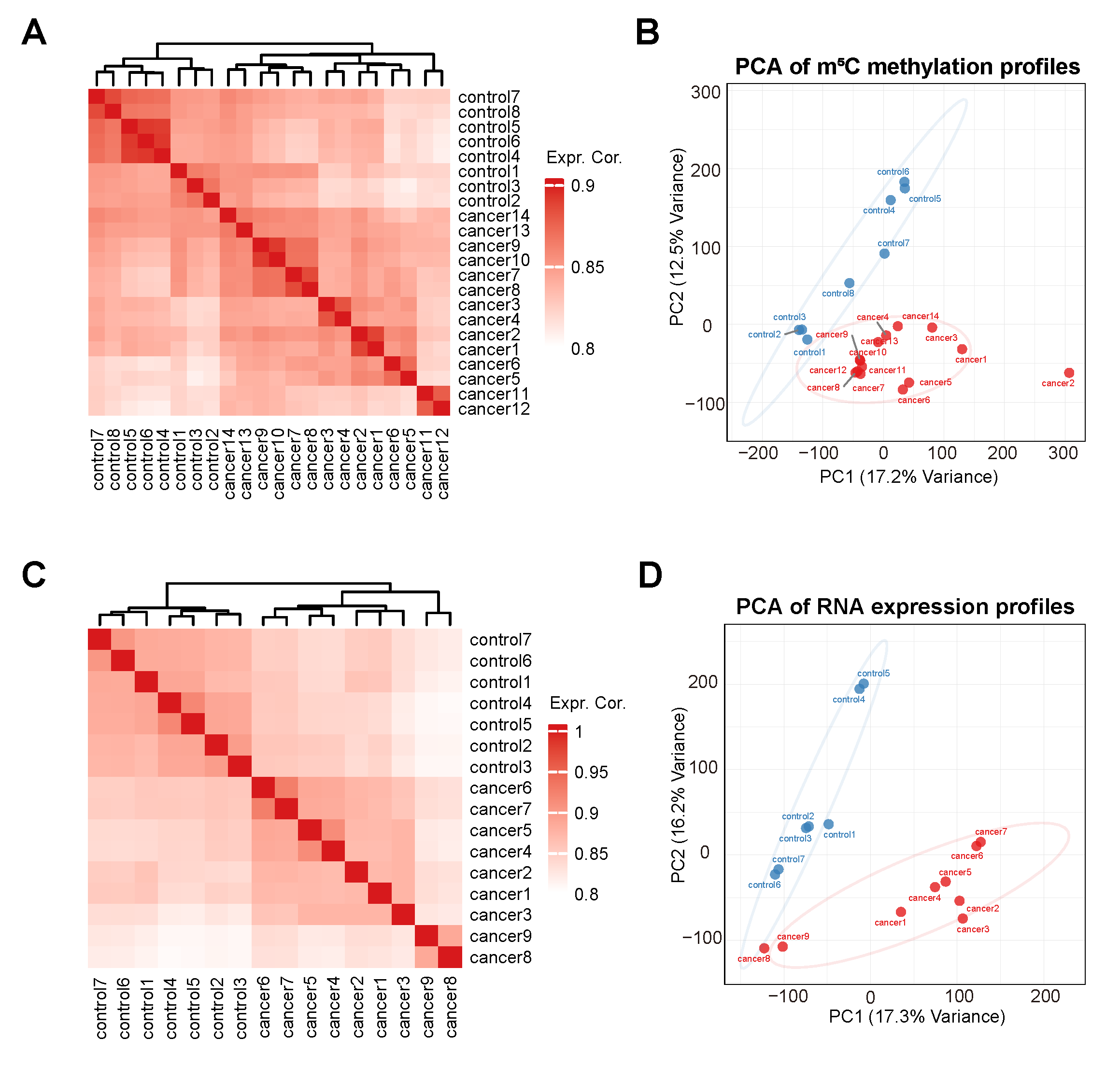
**

**Fig. S1. Correlation and principal component analyses (PCA) reveal high intra-group consistency and distinct molecular signatures in cancer samples.**

(A) Hierarchical clustering heatmap showing Pearson correlation coefficients of m^5^C methylation profiles across all samples. (B) PCA of m^5^C methylation levels separates cancer (red) and control (blue) samples along the first two principal components (PC1: 17.2% variance; PC2: 12.5%). (C) Hierarchical clustering heatmap of RNA-seq expression profiles based on Pearson correlation coefficients across all samples. (D) PCA of RNA expression profiles reveals clear separation between cancer (red) and control (blue) samples along PC1 (17.3%) and PC2 (16.2%).


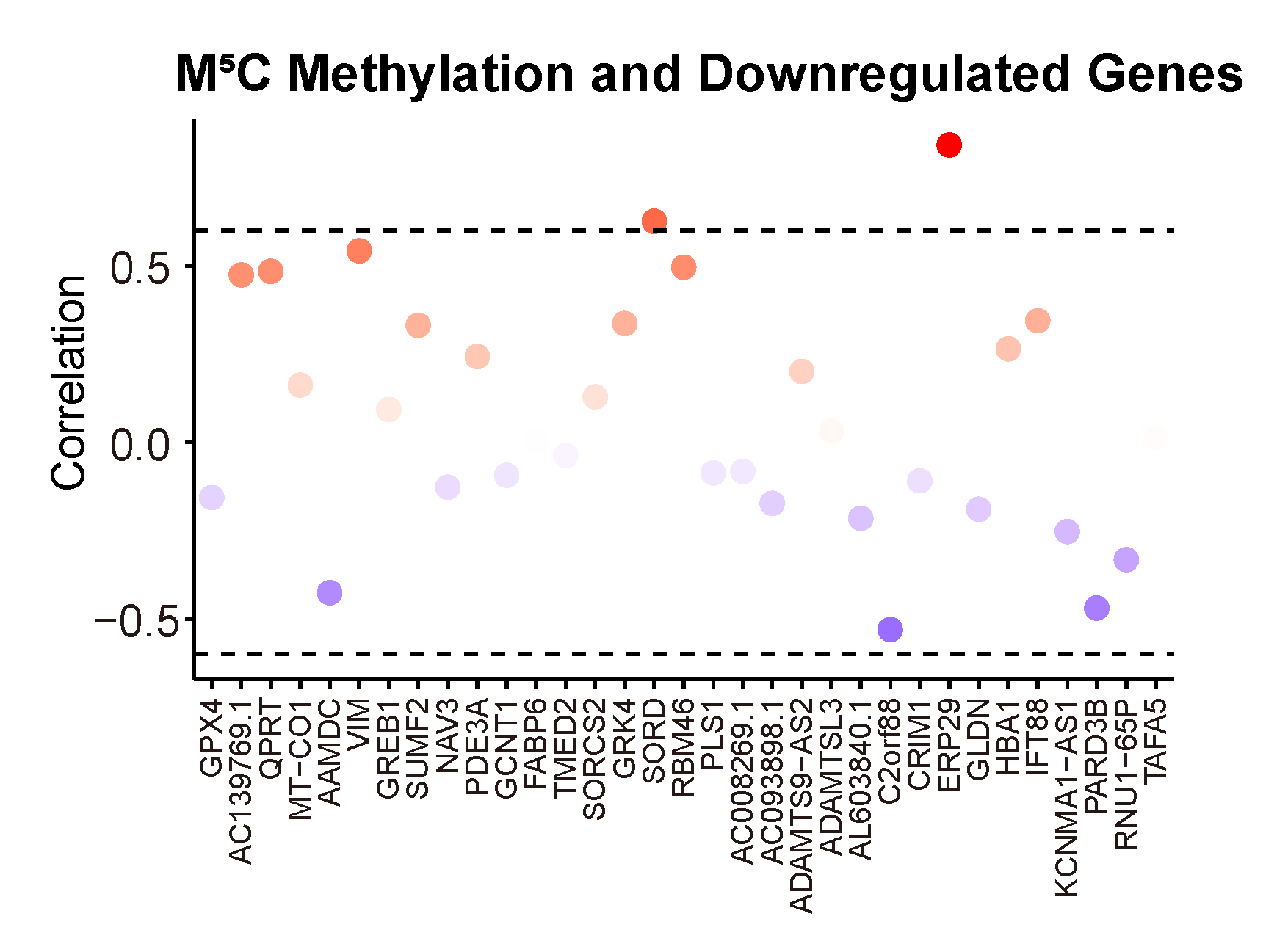


**Fig. S2.** **Correlation between increased m^5^C methylation and downregulated gene expression in cancer.** Correlation plot showing the relationship between m^5^C methylation levels and RNA expression across genes with increased m^5^C methylation and decreased expression in cancer samples. Each dot represents a gene; dot color indicates the strength of the Pearson correlation coefficient.


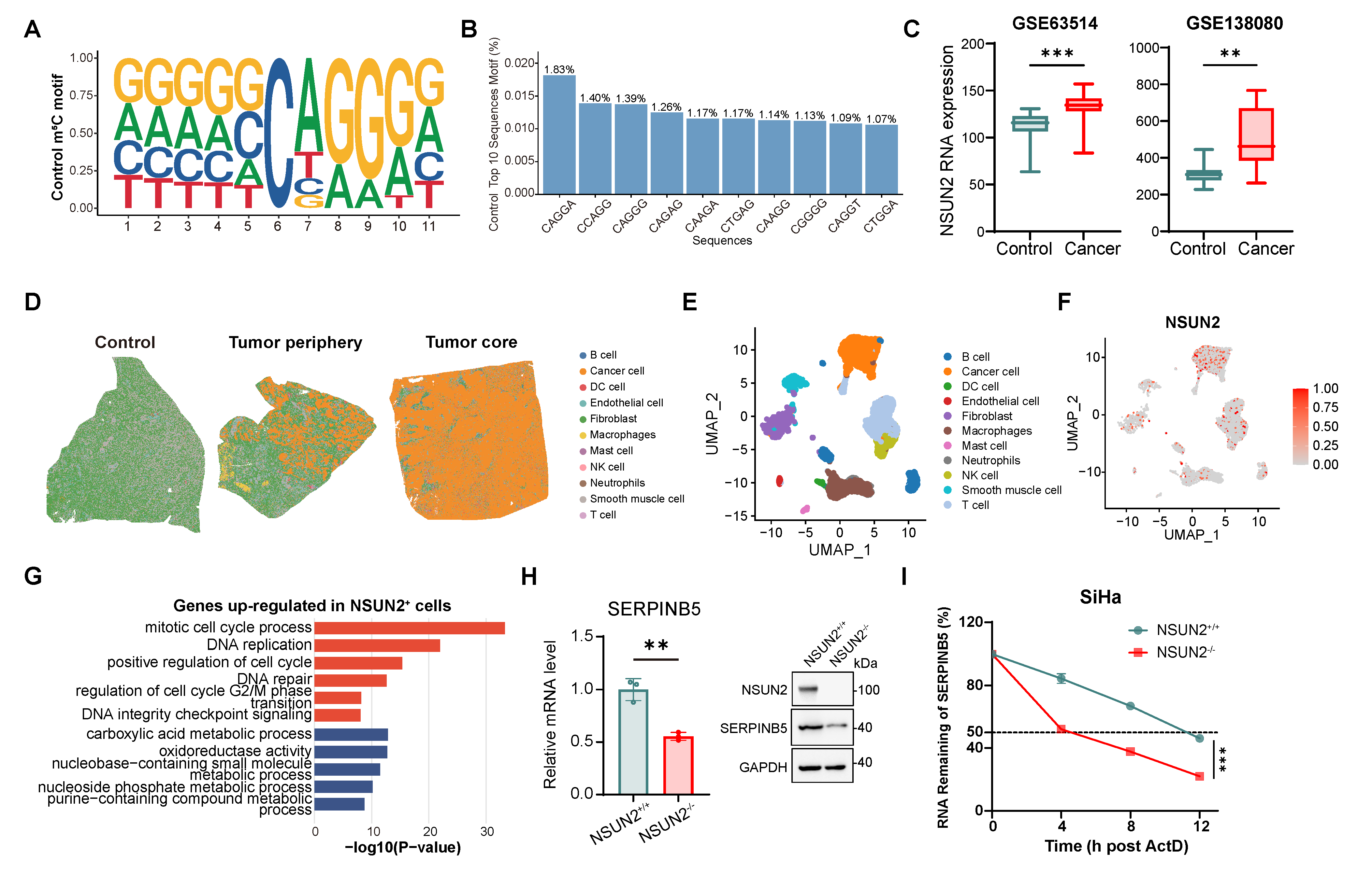


**Fig. S3. NSUN2 expression, sequence specificity, and functional relevance in cervical cancer**

(A) Sequence motif enriched at m^5^C sites identified in control samples. (B) Top 10 enriched sequence contexts flanking control-associated m^5^C sites. The percentage indicates the fraction of total m^5^C sites matching each motif. (C) Box plots of *NSUN2* RNA expression in control and cancer samples from the GSE63514 (n = 24 control, n = 28 cancer) and GSE138080 (n = 10 per group) datasets. Data are presented as box plots showing the median and min to max range. (D) Spatial distribution of cell types in control tissue, tumor periphery, and tumor core based on spatial transcriptomics annotations adapted from Zhou et al., 2024 (52). (E) UMAP plots of cells identified from scRNA-seq data of cervical cancer. Different colors represent distinct cell types. Annotations adapted from Zhou et al., 2024 (52). (F) UMAP plot showing *NSUN2* expression across single cells from cervical cancer samples. (G) Enrichment analysis of genes upregulated in *NSUN2*^+^ cells (vs. *NSUN2*^-^ cells). (H) *SERPINB5* mRNA level (left) (n = 3) and protein level (right) in WT SiHa cells or NSUN2^-/-^ SiHa cells. For qPCR, data are presented as mean ± SD. (I) RNA stability assay of *SERPINB5* mRNA after actinomycin D treatment in WT SiHa cells or NSUN2^-/-^ SiHa cells. Remaining mRNA was quantified by qPCR at the indicated time points (n = 3). Statistical analyses were performed using the two-tailed unpaired t test for (C and H-left), and two-way ANOVA followed by multiple comparisons test for (I). NS, not significant for P > 0.05, *P < 0.05, **P < 0.01, ***P < 0.001.


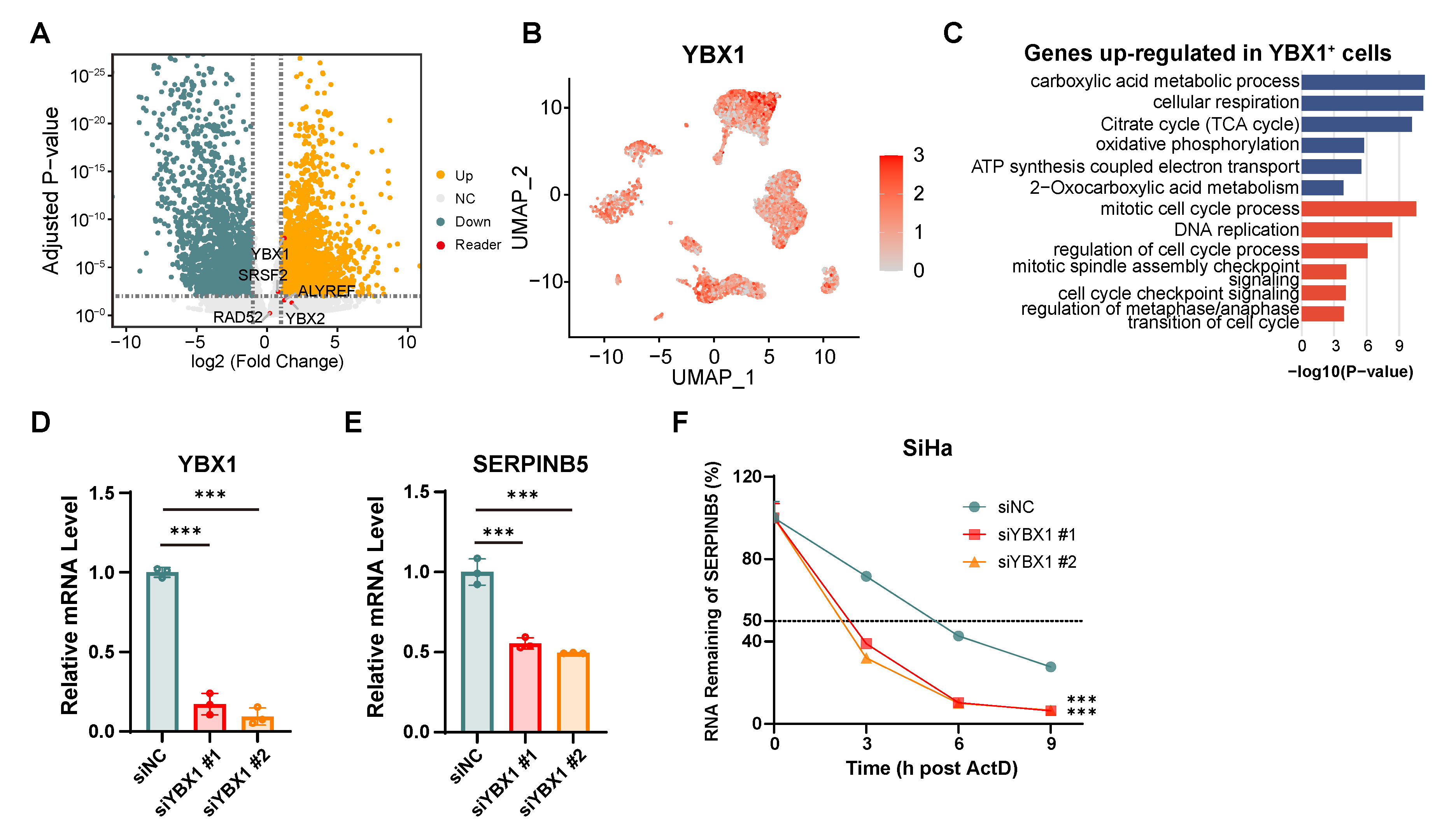


**Fig. S4. Expression profile and regulatory function of YBX1 in cervical cancer cells.**

(A) Volcano plot (P-value and fold change) comparing gene expression differences between cancer and control samples, highlighting m^5^C reader genes (in red). Horizontal dashed line represents adjusted P-value of 0.01, while vertical dashed lines represent log_2_FC = -1 and 1. (B) UMAP plot showing *YBX1* expression across single cells from cervical cancer samples. (C) Enrichment analysis of genes upregulated in *YBX1*^+^ cells (vs. *YBX1*^-^ cells). (D) qPCR analysis of *YBX1* mRNA levels after siRNA knockdown (siYBX1 #1 and siYBX1 #2) in SiHa cells (n = 3). Data are presented as mean ± SD. (E) Relative expression of *SERPINB5* mRNAs following YBX1 knockdown in SiHa cells (n = 3). Data are presented as mean ± SD. (F) RNA stability assay of *SERPINB5* mRNA in SiHa cells treated with actinomycin D following siNC or *YBX1* siRNA knockdown. Remaining mRNA levels were quantified by qPCR at the indicated time points (n = 3). Data are presented as mean ± SD. Statistical analyses were performed using the two-tailed unpaired t test for (D and E), and two-way ANOVA followed by multiple comparisons test for time-course RNA stability assays (F). NS, not significant for P > 0.05, *P < 0.05, **P < 0.01, ***P < 0.001.


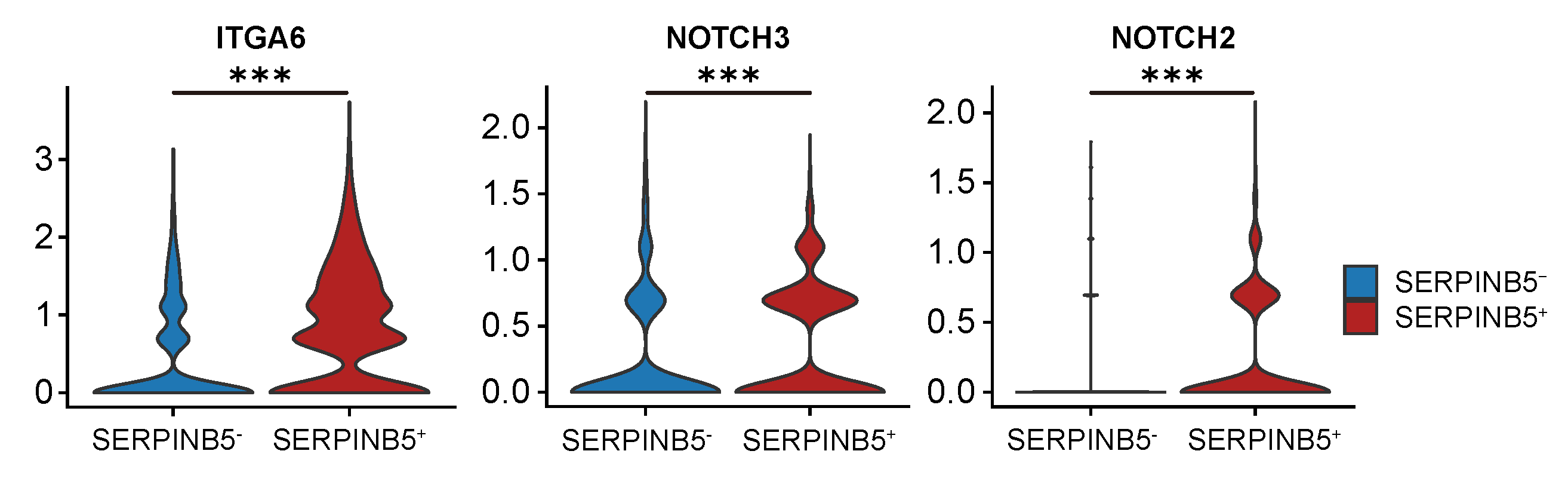


**Fig. S5. Stemness-associated gene expression in SERPINB5^+^ and SERPINB5^-^ cancer cells.** Violin plots showing mRNA expression levels of *ITGA6*, *NOTCH3*, and *NOTCH2* (***P < 0.001, Wilcoxon rank-sum test).


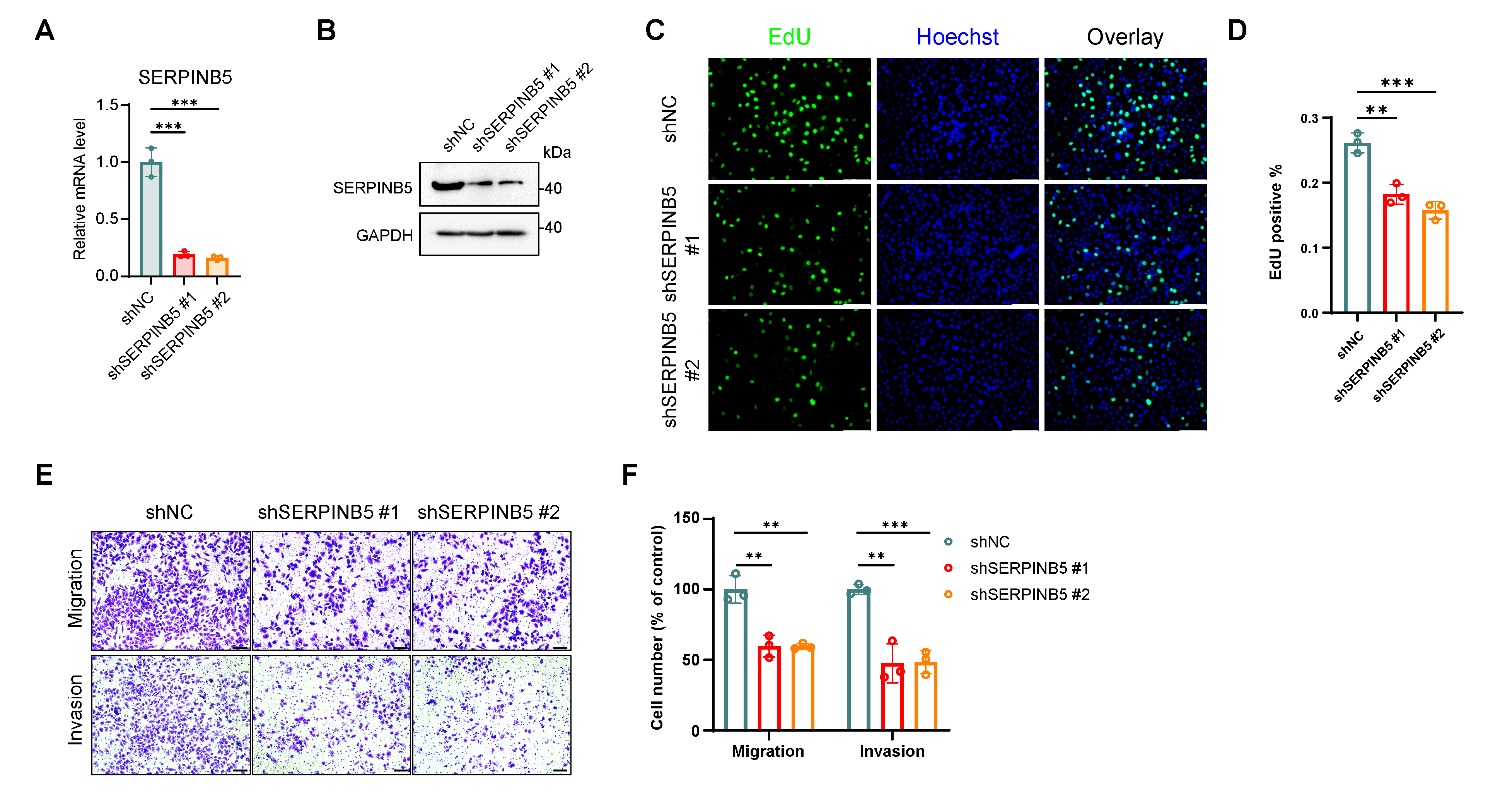


**Fig. S6. SERPINB5 promotes cervical cancer cell proliferation and metastasis in *vitro*.**

(A) qPCR analysis showing *SERPINB5* mRNA levels in SiHa cells transfected with shNC, shSERPINB5 #1, or shSERPINB5 #2. Data are presented as mean ± SD from three independent experiments (n = 3). (B) Immunoblot analysis of SERPINB5 protein levels in SiHa cells shown in panel A. (C) Representative immunofluorescence images showing EdU (green) and Hoechst (blue) staining in SiHa cells transduced with shNC, shSERPINB5 #1, or shSERPINB5 #2. Scale bars, 100 μm. (D) Quantification of EdU-positive cells. Data are presented as mean ± SD from three independent experiments (n = 3). (E) Representative images of crystal violet-stained SiHa cells in transwell migration and invasion assays following SERPINB5 knockdown. Scale bars, 100 μm. (F) Quantification of migrated and invaded cells (n = 3). Data are presented as mean ± SD. Statistical analyses were performed using the two-tailed unpaired t test. NS, not significant for P > 0.05, *P < 0.05, **P < 0.01, ***P < 0.001.


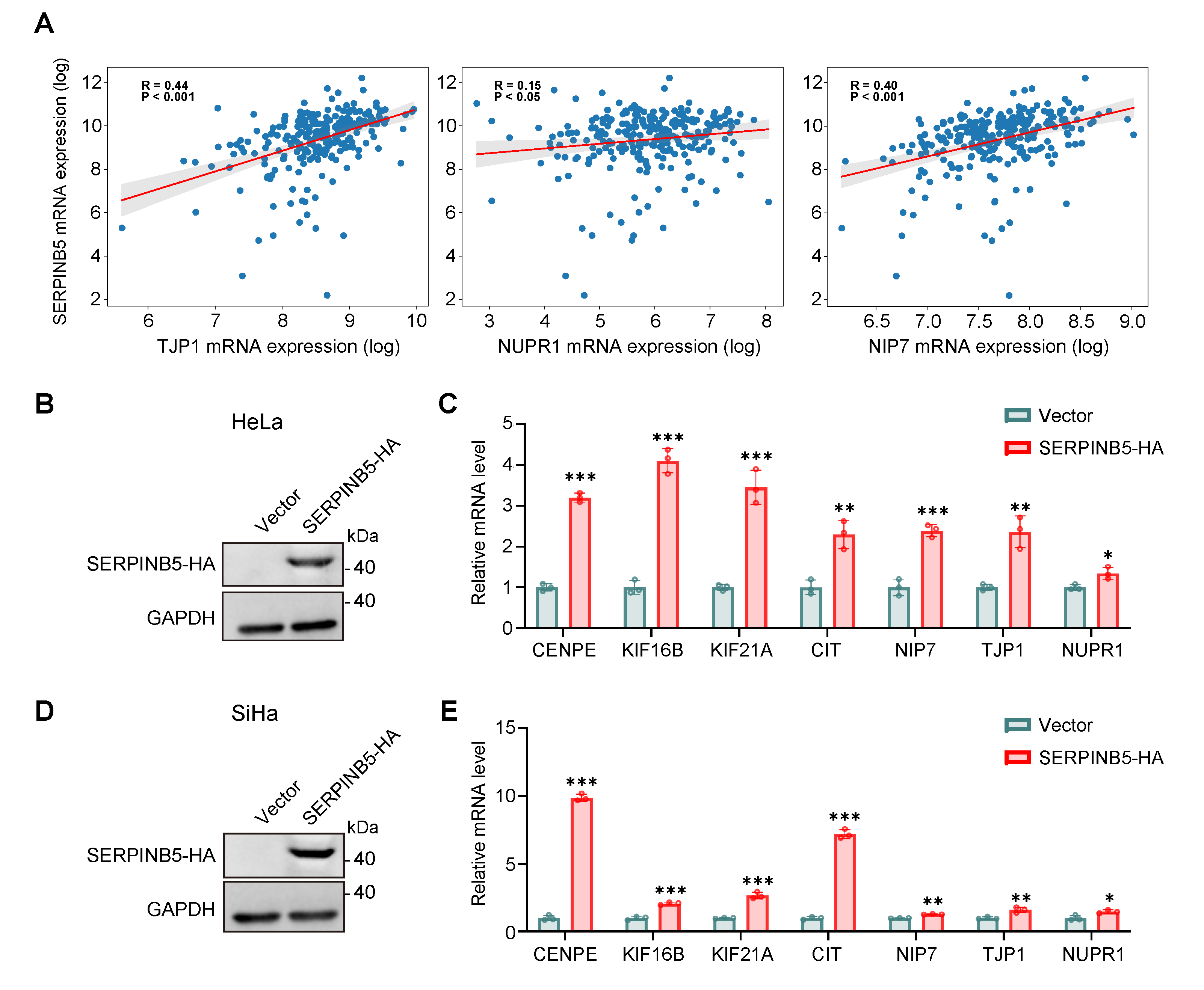


**Fig. S7. SERPINB5 influences the key microtubule and cytoskeletal motor genes.**

(A) Correlation between *SERPINB5* mRNA expression and the expression of *TJP1*, *NUPR1*, and *NIP7* in the TCGA cervical cancer dataset. (B, D) Immunoblot analysis of SERPINB5 protein levels in HeLa (B) and SiHa (D) cells transfected with Vector or SERPINB5-HA plasmid. (C, E) Relative mRNA expression of the indicated genes following SERPINB5 overexpression in HeLa (C) and SiHa (E) cells. Data are presented as mean ± SD (n = 3). Statistical analyses were performed using the two-tailed unpaired t test. NS, not significant for P > 0.05, *P < 0.05, **P < 0.01, ***P < 0.001.


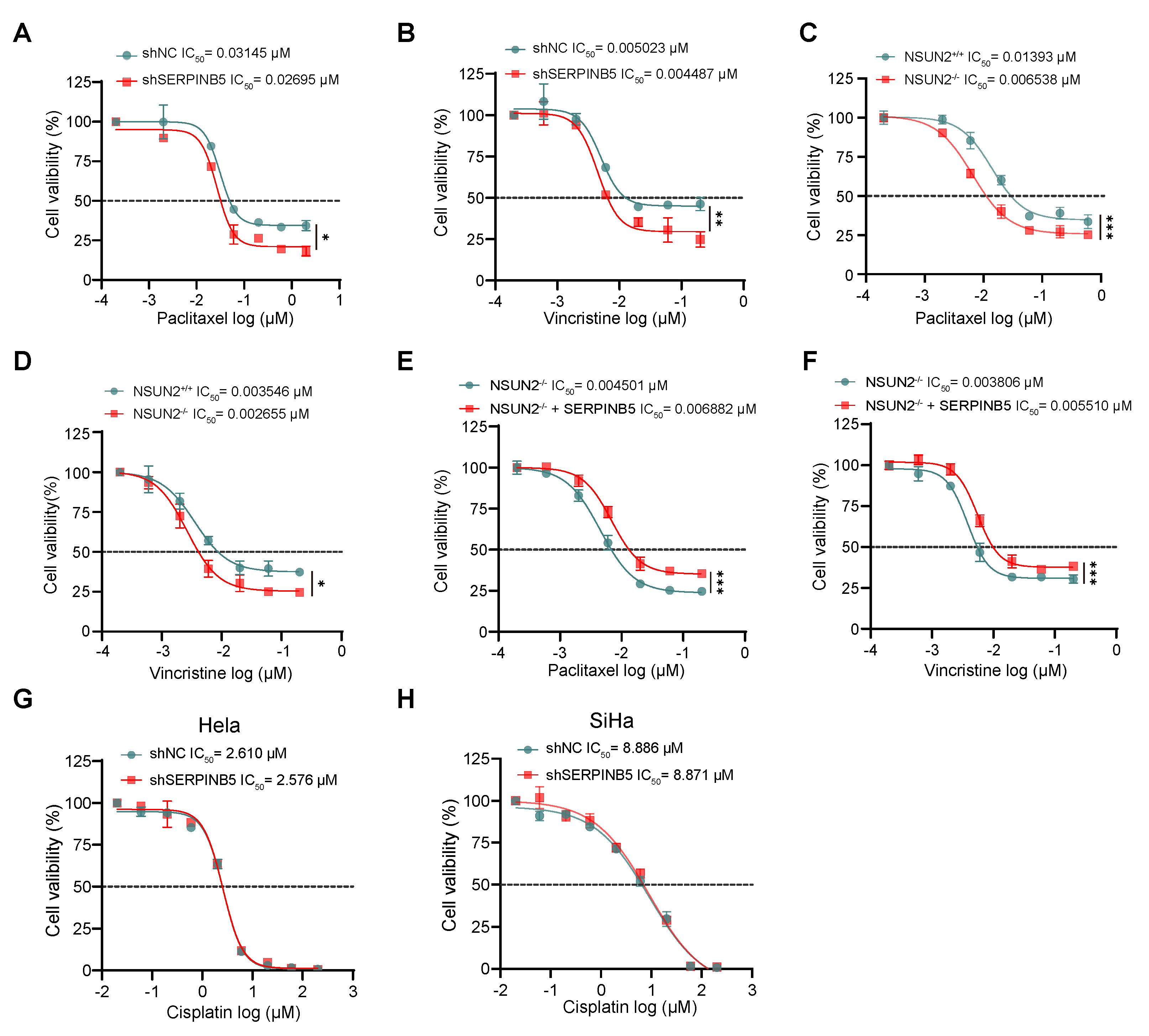


**Fig. S8. SERPINB5 regulates microtubule-targeting chemotherapy response in cervical cancer.**

(A-B) SiHa cells transduced with shNC or shSERPINB5 were treated with paclitaxel (A) or vincristine (B) at gradient concentrations for 48 h, and both cell viability and IC_50_ values were measured using CCK-8 assay (n = 3). (C-D) WT SiHa cells or NSUN2^-/-^ SiHa cells were treated with paclitaxel (C) or vincristine (D) for 48 h, and IC_50_ values were determined by CCK-8 assay (n = 3). (E-F) NSUN2^-/-^ SiHa cells ectopically expressing an empty vector or SERPINB5 were treated with paclitaxel (E) or vincristine (F) at gradient concentrations for 48 h, and cell viability as well as IC_50_ values were measured using CCK-8 assay (n = 3). (G-H) HeLa (G) and SiHa (H) cells transduced with shNC or shSERPINB5 were treated with cisplatin at gradient concentrations for 48 h, and cell viability as well as cisplatin IC_50_ were measured using CCK-8 assay (n = 3). Statistical significance was determined using two-way ANOVA followed by multiple comparisons test. Data are presented as mean ± SD from at least three independent experiments. NS, not significant for P > 0.05, *P < 0.05, **P < 0.01, ***P < 0.001.
