# Supplementary material for: M5C-driven stabilization of SERPINB5 promotes cervical cancer progression and chemotherapy resistance
Source: Cell Death Dis. 2026 Feb 11;17(1):215. doi: 10.1038/s41419-026-08453-2 (PMC12921336; doi:10.1038/s41419-026-08453-2)

**Figure 3H** Figures in Manuscript


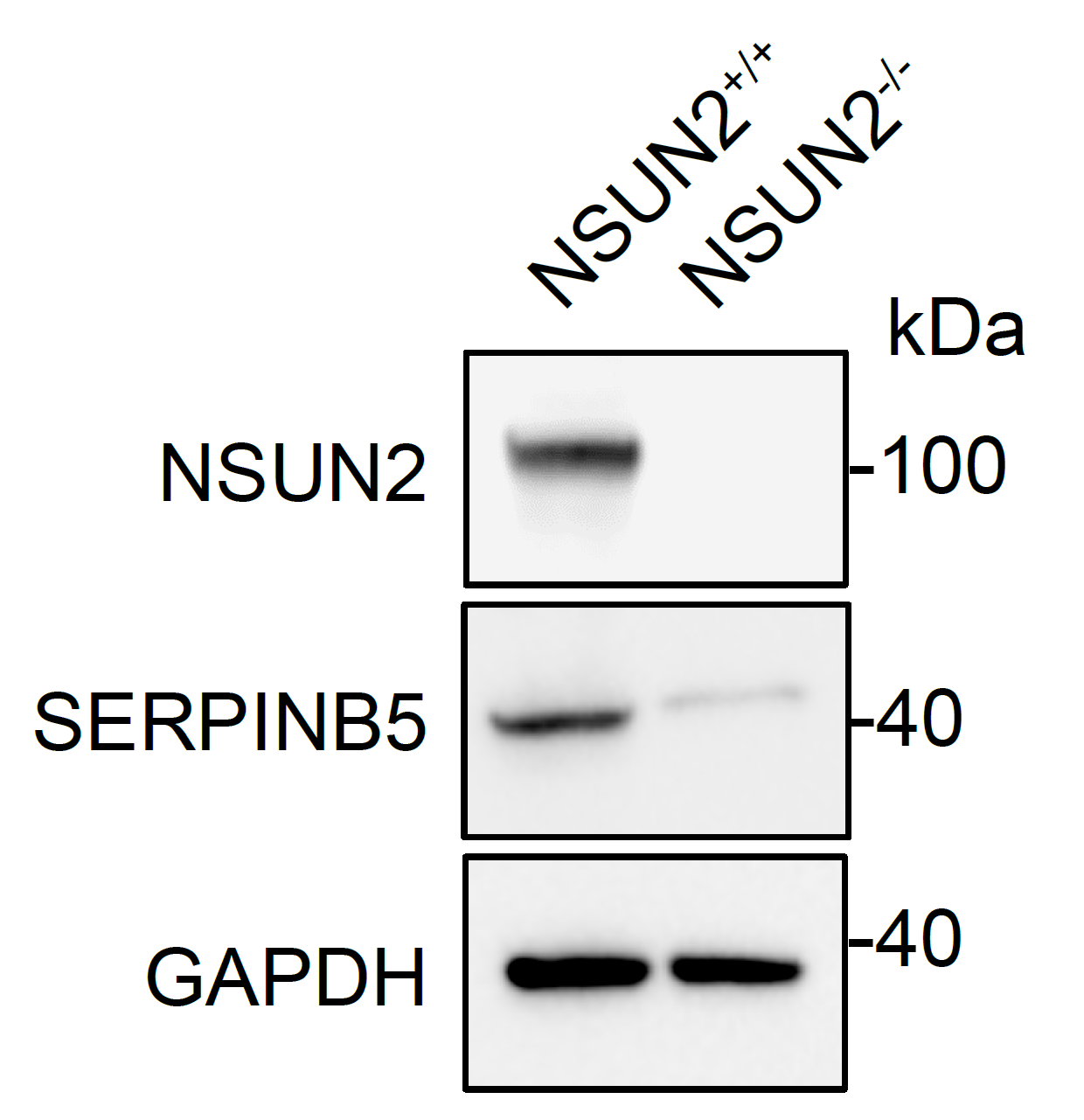


**Figure 3H** Original Source Files


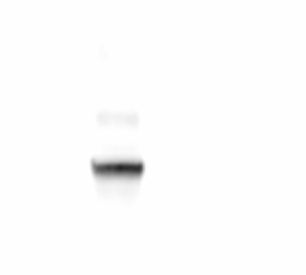
NSUN2 SERPINB5 GAPDH


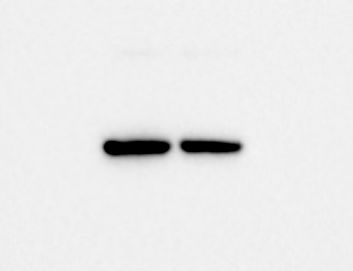

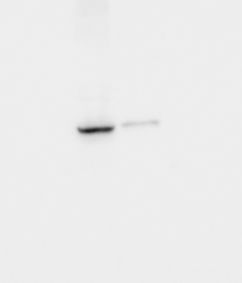


**Figure 3L** Figures in Manuscript


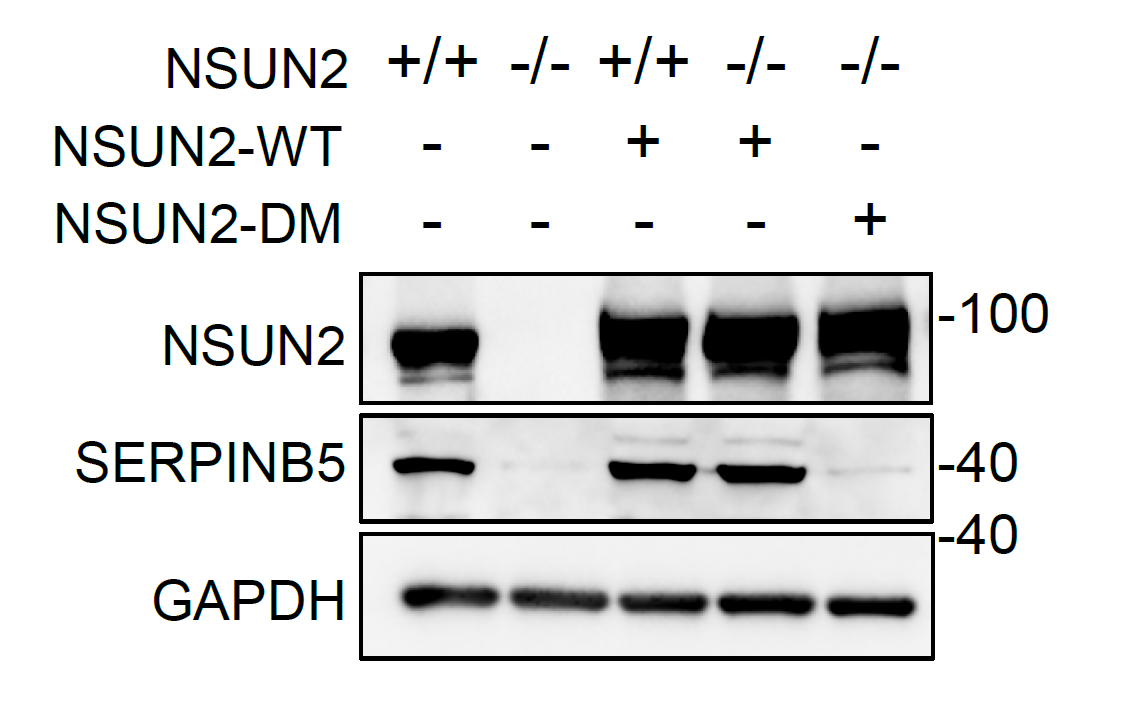


**Figure 3L** Original Source Files


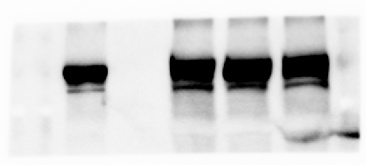
NSUN2 SERPINB5 GAPDH


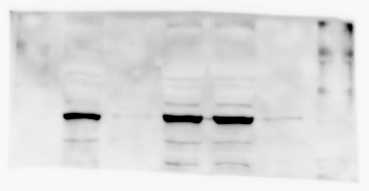

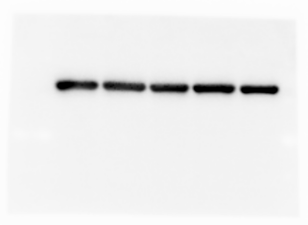


**Figure 4H** Figures in Manuscript


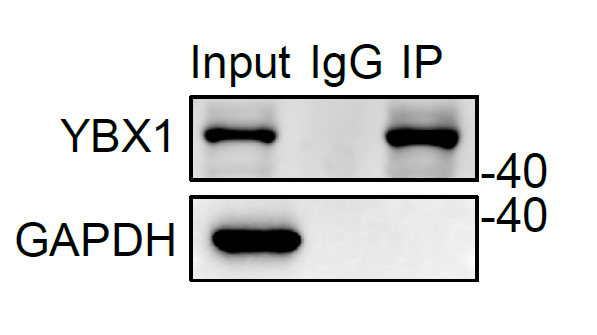


**Figure 4H** Original Source Files

YBX1 GAPDH


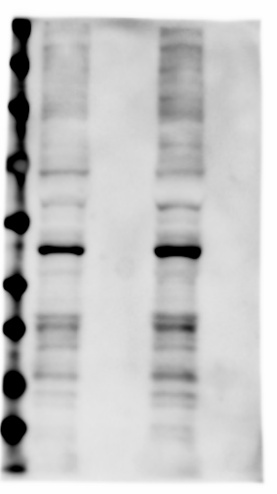

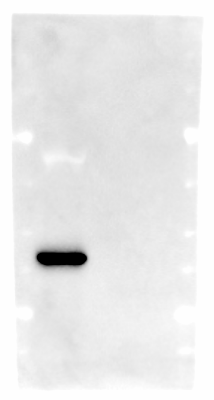


**Figure 5I** Figures in Manuscript


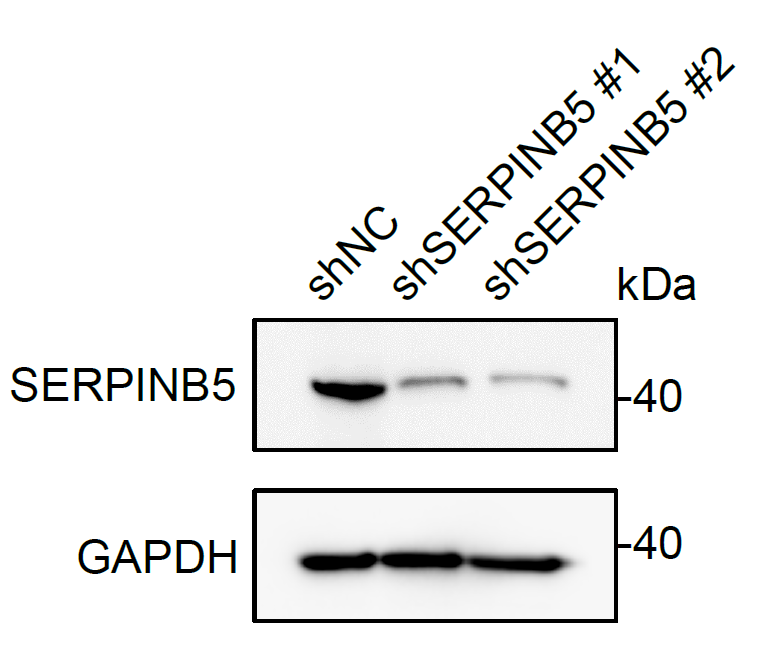


**Figure 5I** Original Source Files

SERPINB5 GAPDH


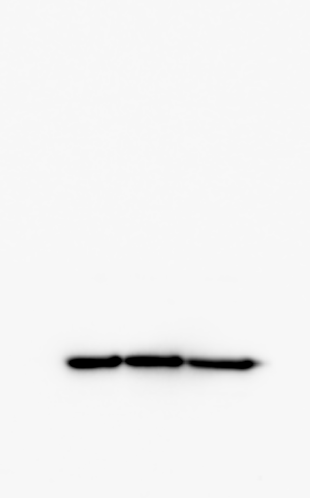

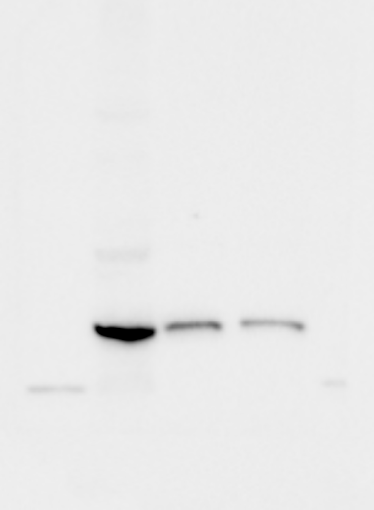


**Figure S3H** Figures in Manuscript


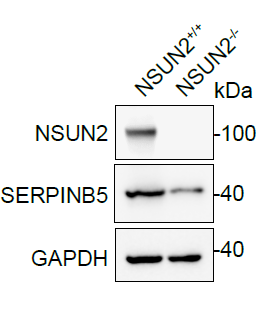


**Figure S3H** Original Source Files

NSUN2 SERPINB5 GAPDH


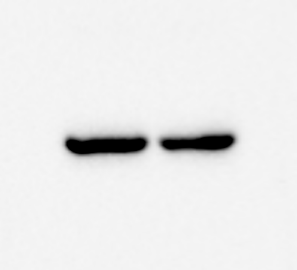

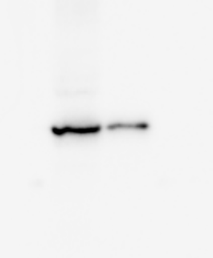

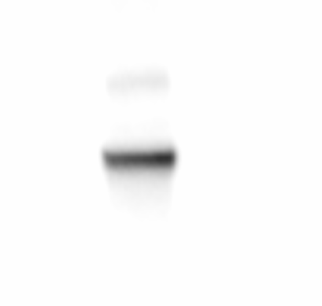


**Figure S6B** Figures in Manuscript


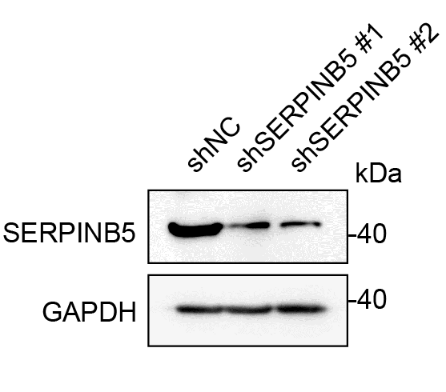


**Figure S6B** Original Source Files

SERPINB5 GAPDH


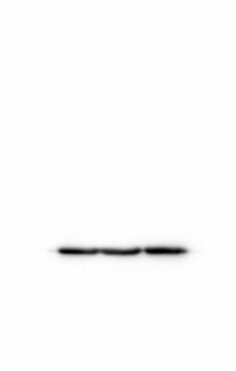

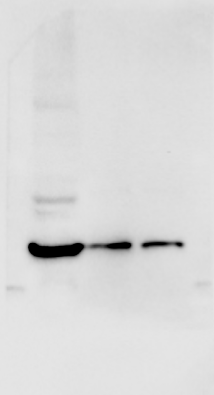


**Figure S7B and D** Figures in Manuscript


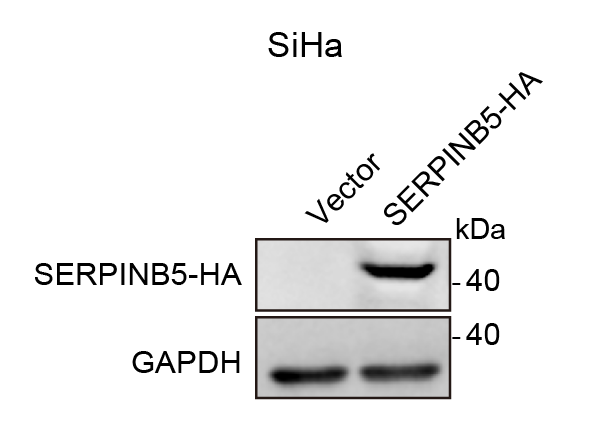

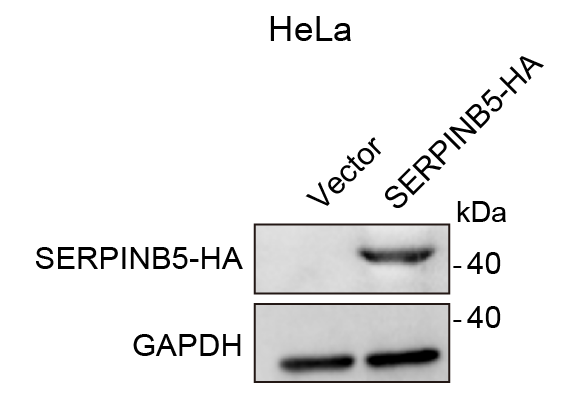
**Figure S7B** **Figure S7D**

**Figure S7B and D** Original Source Files

SERPINB5-HA GAPDH


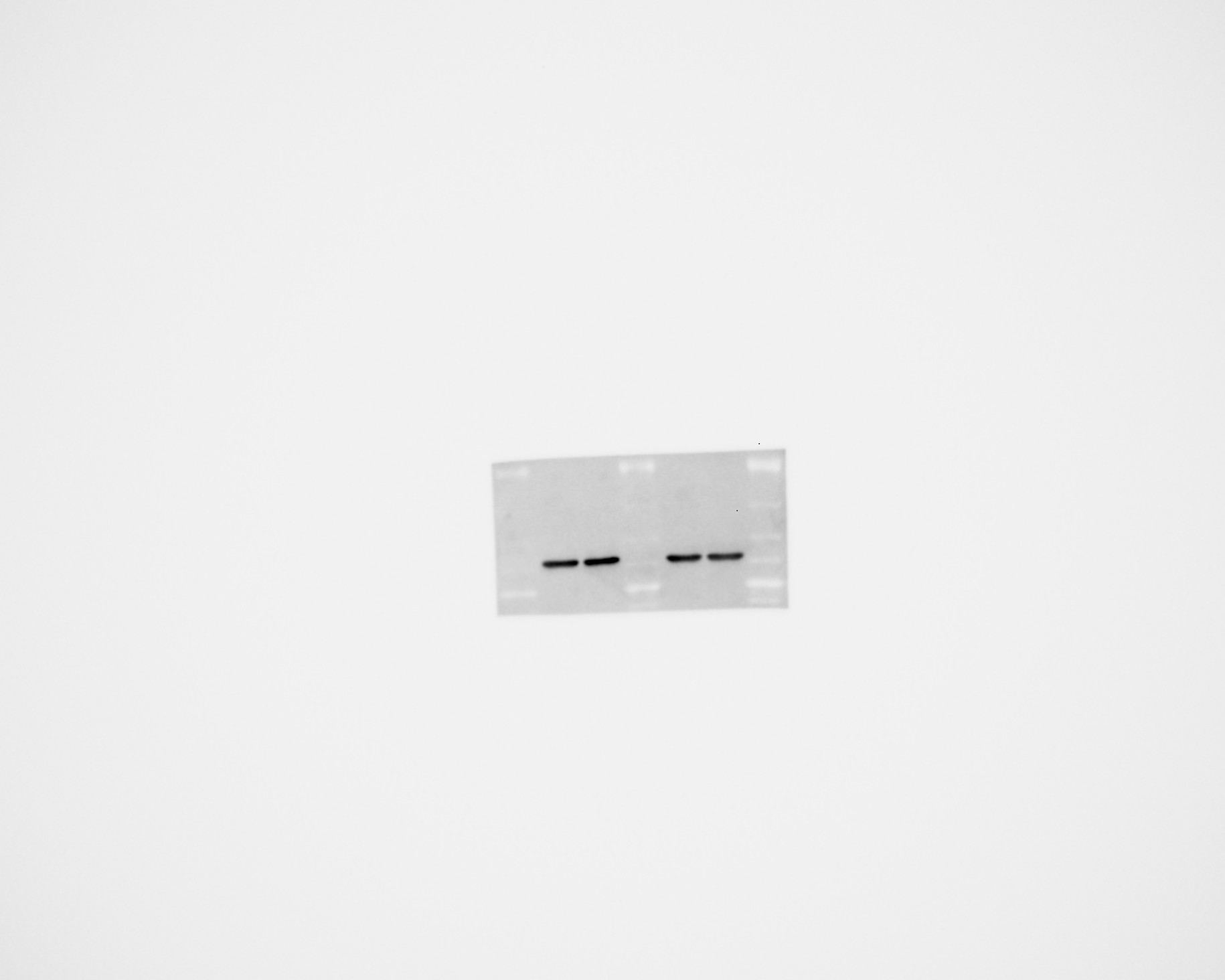

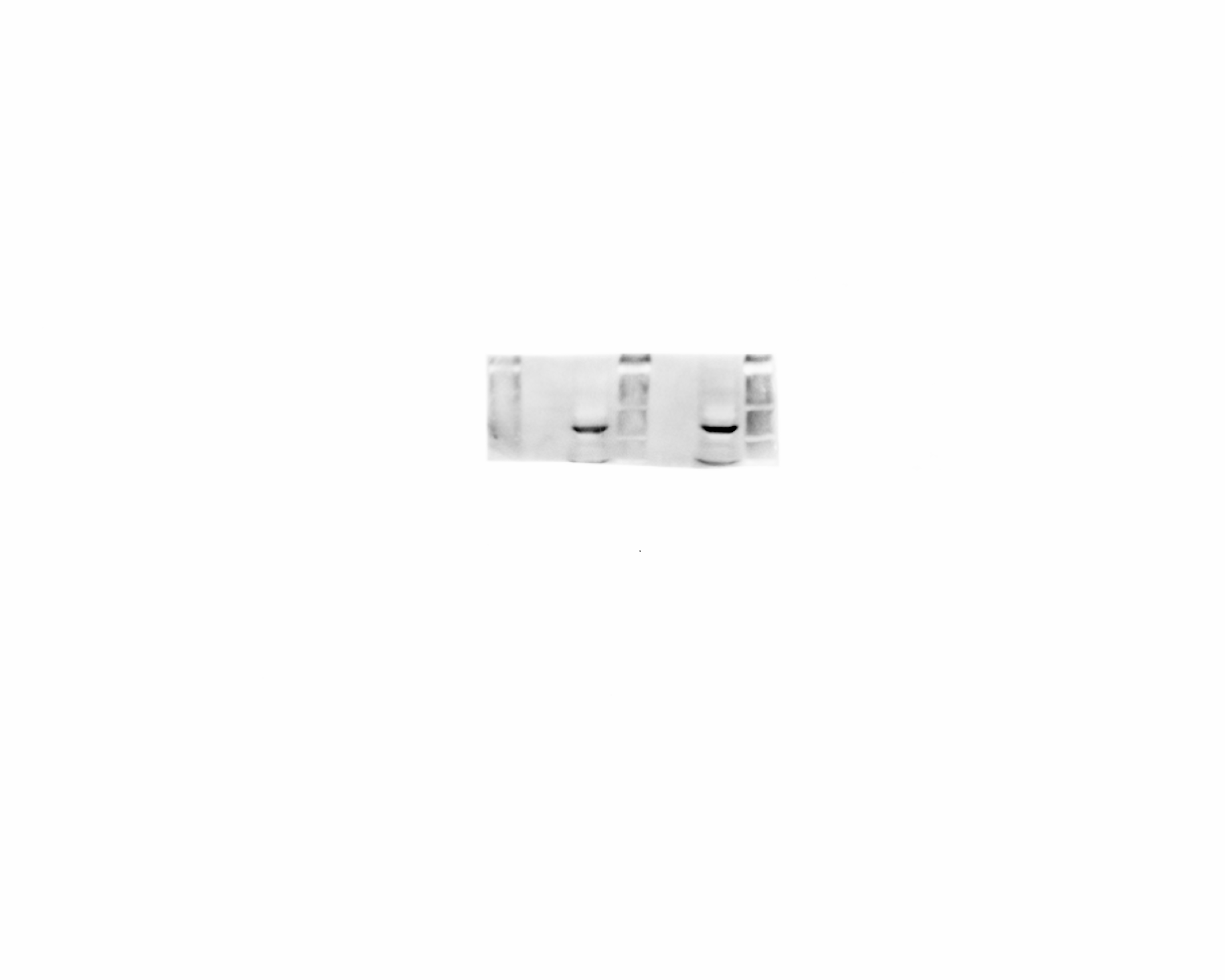

Supplement: Supplementary file 3 — Original Western blots [file 41419_2026_8453_MOESM3_ESM.docx]
